# Middle-temperature thermally stimulated transformations of Cu–Si–O films

**DOI:** 10.1039/d5na01144b

**Published:** 2026-04-24

**Authors:** Oleh Bratus, Antonina Kykot, Mykola Sopinskyy, Pavels Onufrijevs, Oleksandr Gudymenko, Larysa Khomenkova, Volodymyr Yukhymchuk, Tomash Sabov, Oleksandr Oberemok, Anton Semeniuk, Dmytro Kysil, Anatoliy Evtukh

**Affiliations:** a V.E. Lashkaryov Institute of Semiconductor Physics, NAS of Ukraine 41 Pr. Nauky Kyiv 03028 Ukraine o.l.bratus@gmail.com; b Institute of Physics and Materials Science, Faculty of Natural Sciences and Technology, Riga Technical University Paula Valdena Street 7 Riga LV-1048 Latvia; c National University “Kyiv-Mohyla Academy” 2 Skovorody Str. Kyiv 04070 Ukraine; d Educational and Scientific Institute of High Technologies, Taras Shevchenko National University of Kyiv Volodymyrska Street, 64 Kyiv 01601 Ukraine

## Abstract

This paper presents a comprehensive characterization of the structural and optical properties of the Cu–Si–O films prepared by the ion-plasma sputtering method of a combined Cu/Si target in an Ar and O_2_ atmosphere before and after heat treatment at temperatures up to 600 °C. For this purpose, data from seven experimental methods were analyzed: scanning electron microscopy, X-ray photoelectron spectroscopy, dynamic secondary ion mass spectrometry, quantitative X-ray diffractometry, Raman spectroscopy, Fourier transform infrared spectroscopy, and combined reflection–transmission spectroscopy. It was found that as-deposited films represent a uniform over-depth film, the main components of which are suboxides and oxides of silicon and copper with nano- and dispersed inclusions of silicon and copper. At annealing temperatures of 400–600 °C, the film gradually transforms into a heterogeneous structure composed of copper and silicon oxides, CuO and SiO_2_, and inhomogeneity of composition and structure appears along the film depth. The film becomes two-layered, with a denser lower layer and a less dense upper layer depleted in silicon. Similar to substoichiometric silicon oxide (SiO_*x*_) films, the annealed Cu–Si–O films have the best ordering at the level of short and medium range order (minimal value of the Urbach tail energy *E*_U_) after annealing at ∼600 °C. The obtained results demonstrate the possibility of deliberately tuning the structure and physicochemical properties of Cu–Si–O films over a wide range, making them promising for applications in sensing, dielectric layers for microelectronics, and functional oxide coatings.

## Abstract 2

Annealing transforms a uniform Cu–Si–O film into a bilayer structure through thermally driven redistribution of copper, silicon, and oxygen. This process leads to pronounced microstructural ordering and changes in optical properties.

## Graphical abstract

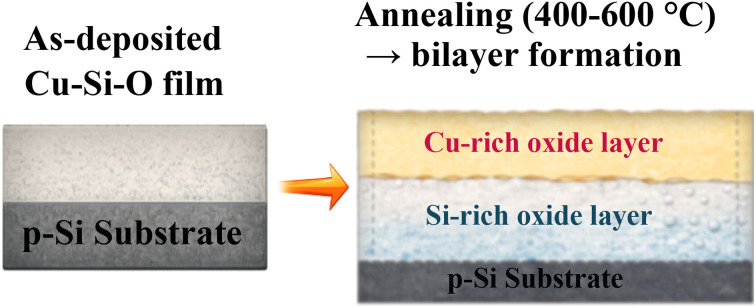

## 1 Introduction

Research into materials based on or incorporating the Cu–Si–O system is of considerable interest due to their broad application potential. Understanding Cu–Si interactions and interface formation is crucial for electronics and semiconductor technologies.^1^ As silicon-based technologies continue to dominate integrated circuit manufacturing, the Cu–Si system has attracted particular attention for microelectronics,^2^ catalysis,^3^ sensors,^4^ lithium-ion batteries,^5^ solar cells,^6^ and other related devices. Copper is an important material in electronics because of its high electrical conductivity, affordable price, and environmental safety. However, its use in nanoelectronics faces several challenges, particularly the tendency of copper to diffuse into silicon substrates, which can cause leakage currents or short circuits, as well as its susceptibility to oxidation even under vacuum conditions. To reduce these effects, dielectric barriers such as SiO_2_ are often used, as they are insulating, readily fabricated, and show good stability and adhesion to copper.^7^

Another challenge is copper oxidation. In Cu–O systems, both Cu_2_O and CuO can be formed depending on processing conditions.^8^ Cu_2_O and CuO are intensively studied p-type semiconductors relevant to optoelectronics and energy conversion,^9–13^ and Cu-based nanostructures are also attractive for functional coatings and composite materials. At the nanoscale, Cu tends to agglomerate and oxidize, which can degrade the functional performance of the films; therefore, encapsulation in silica-based matrices is often used to enhance dispersion and improve long-term stability.^14–16^ Post-deposition thermal treatments are frequently required to improve film quality and to stimulate structural transformations;^17^ however, in Cu–Si–O systems they may also induce silicide formation, which can strongly modify transport and stability.^18^ Copper silicide formation can also affect oxidation behavior and electrical properties.^5^

In parallel, non-stoichiometric SiO_*x*_ and stoichiometric SiO_2_ thin films have been thoroughly studied^19–22^ and are widely used in semiconductor microelectronics^23–26^ and related technologies. Depending on the ratio of the metallic inclusions to the dielectric matrix, the structural, optical, and electrical properties of such nanocomposite systems can vary widely.^27–29^

The ability of copper to form oxygen-containing compounds in which Cu exhibits oxidation states of +1 or +2, its propensity to form a series of Cu_*x*_Si_*y*_ intermetallic compounds with silicon, and the tendency of silicon suboxides SiO_*x*_ (*x* < 2) to disproportionate with the formation of nc-Si–SiO_*y*_ (*y* > *x*) nanocomposites suggest that, by varying the composition and synthesis conditions of Cu–Si–O system materials, it is possible to obtain (nano)composites whose structure and functional properties can be tuned over a very wide range. At the same time, achieving Cu–Si–O-based materials with predetermined properties requires further detailed studies of the formation processes as a function of composition and processing conditions; the present work contributes to addressing this challenge.

Most of the literature on Cu/SiO_2_-based nanocomposites relies on embedding or encapsulating pre-defined Cu morphologies (flakes, nanowires, and nanoparticles) in a silica matrix.^15,16^ In contrast, the novelty of the present fabrication approach lies in depositing an initially single-layer Cu–Si–O film by ion-plasma sputtering (IPS)^30,31^ and using controlled post-deposition annealing to trigger thermally stimulated vertical redistribution of components, resulting in a bilayer-like architecture with distinct near-surface and near-substrate regions.

Similar annealing-driven Cu out-diffusion and microstructural rearrangement have been reported for co-sputtered Cu–SiO_2_ composites.^32^ Related A–B–O (A, B = metals) thin-film systems can also exhibit annealing-induced vertical separation; for example, co-sputtered Cu(Ti) films on SiO_2_ undergo phase separation upon annealing, with Ti accumulating near the SiO_2_ interface and Cu enriching the near-surface region, eventually forming a continuous Cu-rich layer.^33^ However, to the best of our knowledge, an explicit and well-resolved bilayer-like stratification driven by middle-temperature post-deposition annealing has not been previously reported for mixed Cu–Si–O films comprising both oxide phases and elemental inclusions.

Here, we provide direct experimental evidence for a stable bilayer-like configuration with distinct near-surface and near-substrate regions. The structure and composition of the films were analyzed using a number of advanced methods: scanning electron microscopy (SEM), X-ray diffraction (XRD), X-ray photoelectron (XPS) spectroscopy, Auger spectroscopy, secondary ion mass spectrometry (SIMS), Raman spectroscopy, Fourier transmission infrared (FTIR) spectroscopy, and optical reflection and transmission spectroscopy.

## 2 Experimental

**Fig. 1:**
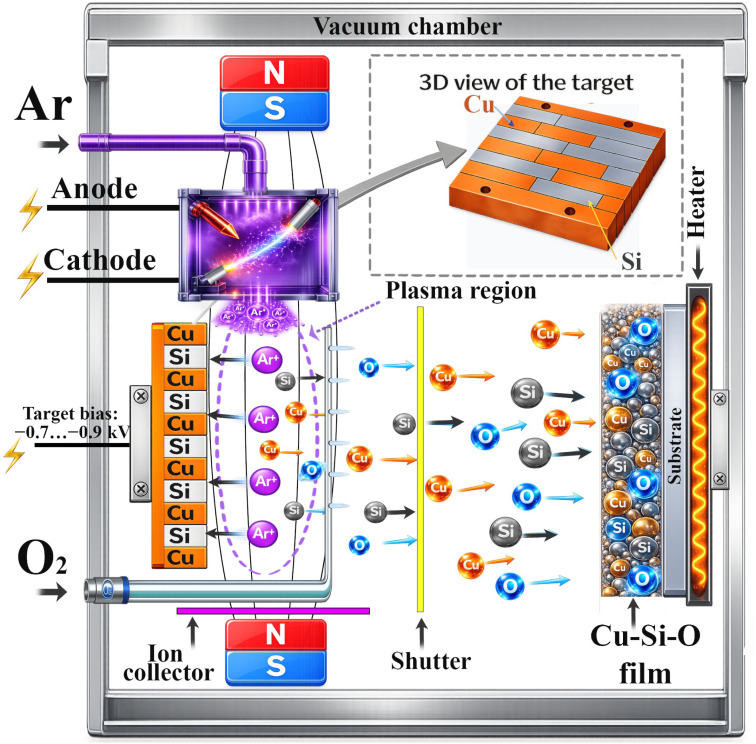
Schematic illustration of ion-plasma sputtering (IPS): reactive sputtering of a combined Cu–Si striped target (3D view shown) in an Ar/O_2_ discharge, transport of sputtered species through the plasma region, and deposition on a heated substrate forming a Cu–Si–O film.

To form Cu–Si–O films, the ion-plasma sputtering (IPS) method was used, which allows sputtering both semiconductor and metal targets in a working atmosphere of an Ar/O_2_ gas mixture (Fig. 1). This method is based on the use of a non-independent arc gas discharge between a thermionic cathode and anode, which forms a dense electron–ion plasma at low operating voltages (tens of volts) and a pressure of the order of 10^−4^ to 10^−5^ torr. The sputtering target is placed in the plasma region as a third electrode and a negative bias (about 1–3 kV) is applied to it relative to the anode, which ensures intensive bombardment of the target with positive plasma ions and its sputtering. Particles of the sputtered material (mainly neutral atoms and partly ions) are transported to the substrate and deposited on it, forming a thin film (the target–substrate distance was 180 mm). Before supplying the working gases, the chamber was pumped to the base pressure *P*_base_ = 1.0 × 10^−6^ torr. To form oxide films, oxygen is additionally supplied to the chamber; the sputtered atoms interact with activated oxygen forms (mainly O, O^+^ and O_2_^+^) in the plasma and/or on the substrate surface, which leads to their partial or complete oxidation and deposition of the material in the form of an oxide. The presence of a mechanical shutter allows preliminary ion cleaning (target conditioning) of the target before deposition.

For the simultaneous sputtering of silicon and copper, a combined square target of 100 × 100 mm was designed and manufactured, formed by alternating rectangular Cu and Si strips of 10 × 100 mm. The Cu–Si target was mounted on a single holder and a negative target voltage was applied to it, so it operated as one sputtering electrode (single-cathode configuration). Simultaneous sputtering is ensured by the fact that ions from the same plasma simultaneously bombard the open Cu and Si strips of the combined target. Since the sputtering yields of Cu and Si under the same ion bombardment are different, the ratio of the areas of the components of the combined target (Cu/Si) was used as the main parameter for adjusting the relative fluxes of Cu and Si and was controlled according to the results of post-deposition composition analysis. Adjusting the Ar/O_2_ ratio in the working atmosphere additionally allows controlling the degree of oxidation of the components, and therefore, varying the elemental and phase composition of the formed Cu–Si–O films. The films were deposited on p-type Si(100) substrates with resistivity *ρ* = 10 Ω cm and thickness 525 ± 15 µm, and the combined Cu–Si target was assembled using Si strips of the same p-type Si(100) material together with Cu strips cut from a T2 copper sheet (GB/T 5231), *i.e.*, Cu-ETP copper (UNS C11000, EN CW004A; Cu ≥ 99.90 wt% and O ≤ 0.040 wt%).

The deposition parameters were as follows: the working (process) pressure *P*_work_ = 6.5 × 10^−4^ torr, substrate temperature *T*_dep_ = 100–120 °C, cathode heating current *I*_K_ = 144 A, anode voltage *U*_A_ = 50 V, anode current *I*_a_ = 10 A, target voltage *U*_t_ = 0.7–0.9 kV, and target current *I*_t_ = 0.6 mA. The Cu/Si target area ratio was 1/1, the working gas ratio in the chamber during deposition was O_2_/Ar = 10/55 = 0.18, and the deposition time was *t* = 10 min.

To study the effect of the annealing temperature, *T*_ann_, on the structural and optical properties of Cu–Si–O films, we examined the initial structures and those that were annealed in an argon atmosphere for 30 minutes at *T*_ann_ = 400, 600 °C.

The morphological structure of the films and their thickness were examined with SEM. For this, a Tescan MIRA 3 LMU scanning electron microscope was used. A Gatan 682 PECS system was used for sample preparation.

The depth distribution of elements in the films was investigated by dynamic SIMS (D-SIMS) using an ATOMIKA 4000 quadrupole instrument (PerkinElmer, Germany). The element depth profiles were obtained using a 200 nA O_2_^+^ primary ion beam with an energy of 5 keV. The angle of incidence of the beam on the normal to the surface was 0°. The lateral square raster of the crater was 500 µm × 500 µm. The analyzed area was 9% in the center of the formed crater.^34^

XPS measurements were performed using a PHI 5600 spectrometer with monochromatic Al Kα radiation (1486.6 eV). Survey and high-resolution spectra were acquired with pass energies of 93.9 eV and 11.75 eV, respectively. Prior to analysis, the samples were sputter-cleaned for 2 min using Ar^+^ ions (5 keV), and charge neutralization was applied. The atomic percentage of the as-deposited (unannealed) film, determined by XPS, was as follows: Cu – 23.4 at%, Si – 24.7 at%, and O – 51.9 at%.

Structural studies were carried out with the X-ray diffraction method (XRD) using a PANalytical X'Pert PRO – MRD diffractometer with Cu Kα_1_ radiation (*λ* = 0.15406 nm). XRD patterns were recorded in grazing-incidence geometry with an incidence angle of 1.5°.^35^

Fourier transform infrared (FTIR) spectra of the films were measured in the 400–4000 cm^−1^ spectral range using a PerkinElmer Spectrum BX FTIR spectrometer. Clean virgin silicon plates were used as the reference.^36,37^

Raman scattering spectra were registered using an MDR-23 spectrometer equipped with a cooled CCD detector (iDus 401 A Andor). As the excitation source, a diode-pumped 457 nm solid-state laser was used. The laser power density on the sample surface was below 10^3^ W cm^−2^ to preclude any photothermal modification of the samples. The spectral resolution was 3 cm^−1^. It was estimated based on the detected width of the Si TO phonon peak in the bulk Si substrate (centered at 520.5 cm^−1^).^38^

Transmittance *T* and reflectance *R* at normal and near normal (11°) incidence angles, respectively, were measured in the spectral range of 185–1200 nm using a double-beam spectrophotometer Specord 210 plus. The radiation sources were a deuterium lamp (in the ultraviolet region of the spectrum) and a halogen lamp (in the visible region of the spectrum). Switching of radiation sources was carried out at 320 nm. The slit width was 1 nm.

## 3 Results and discussion

### 3.1. Investigation of the surface and cross-sectional morphology of the Cu–Si–O films using SEM

**Fig. 2:**
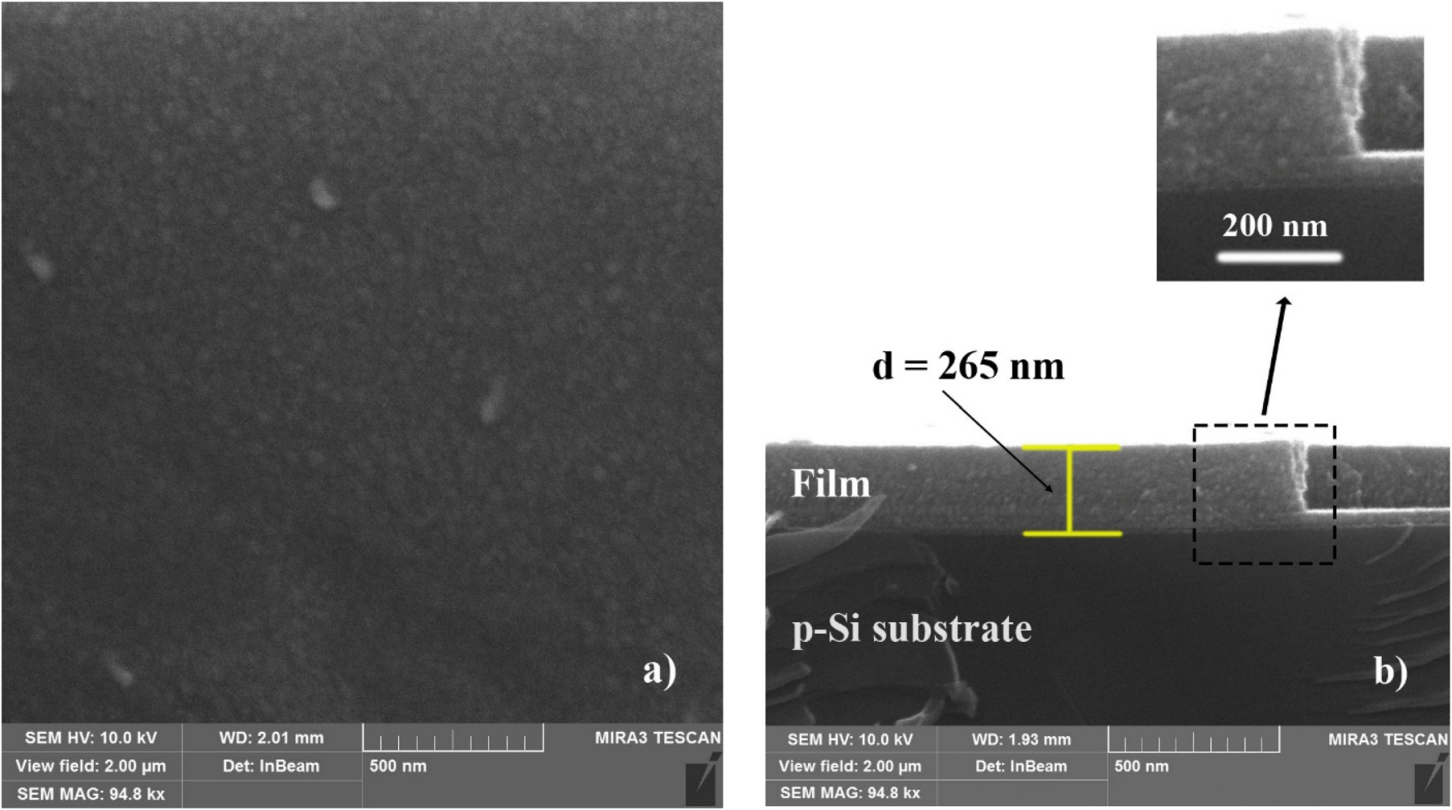
Surface (a) and cross-sectional (b) SEM images of the as-deposited Cu–Si–O film deposited on a p-Si(100) substrate.

The results of SEM observation of the initial (unannealed) Cu–Si–O film are presented in Fig. 2: surface (a) and cross-section (b). The SEM image of the film surface reveals a mosaic-like morphology with a statistically uniform distribution of dark and bright regions and characteristic lateral feature sizes below 50 nm (see Fig. 2a). The surface exhibits a uniform granular structure with nanoscale contrast variations.

The cross-sectional image shows a continuous film with nearly uniform contrast throughout the entire thickness, *d* = 265 nm (see Fig. 2b).

**Fig. 3:**
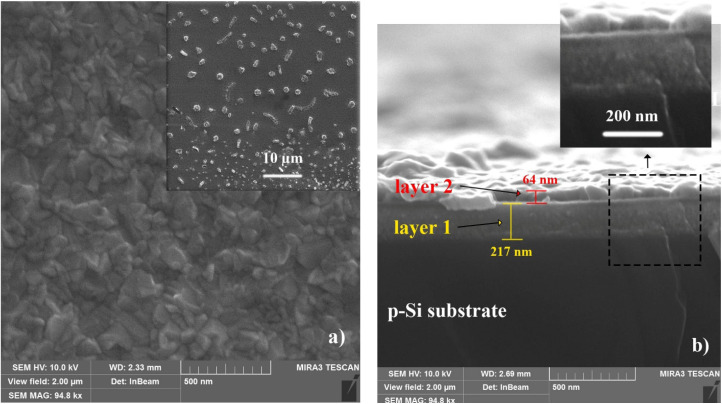
Surface (a) and cross-sectional (b) SEM images of the Cu–Si–O film on p-Si(100) after annealing at 400 °C (Ar, 30 min). The inset in (a) shows a lower-magnification surface view (scale bar: 50 µm). The cross-sectional image reveals a bilayer film (layers 1 and 2).

Fig. 3 shows SEM images of the Cu–Si–O film annealed at 400 °C: surface (a) and cross-section (b). Annealing leads to the formation of uniformly distributed isolated island structures on the surface, ranging from rounded shapes with an average diameter of *D* = 0.5–1.5 µm to elongated shapes with an average length of *l* = 2.3–4.5 µm. The cross-sectional image indicates that annealing at 400 °C results in the formation of two layers with different thicknesses and compositions (indicated by varying contrast), with thicknesses of *d*_1_ = 217 nm and *d*_2_ = 64 nm. Comparing the cross-sectional images of the as-deposited sample and the sample that was annealed at 400 °C reveals a slight increase in the total film thickness from 265 nm to 281 nm (sum of the two layers) (see Fig. 3b). Notably, the bottom layer remains homogeneous, similar to that of the original film.

The appearance of two distinct layers after annealing is attributed to annealing-driven redistribution of constituents and partial phase separation in the Cu–Si–O film, leading to regions with different local density and/or composition and, consequently, different SEM contrast. This interpretation is further supported by the XPS and SIMS results discussed below, which provide insight into the mechanisms underlying the bilayer formation.

**Fig. 4:**
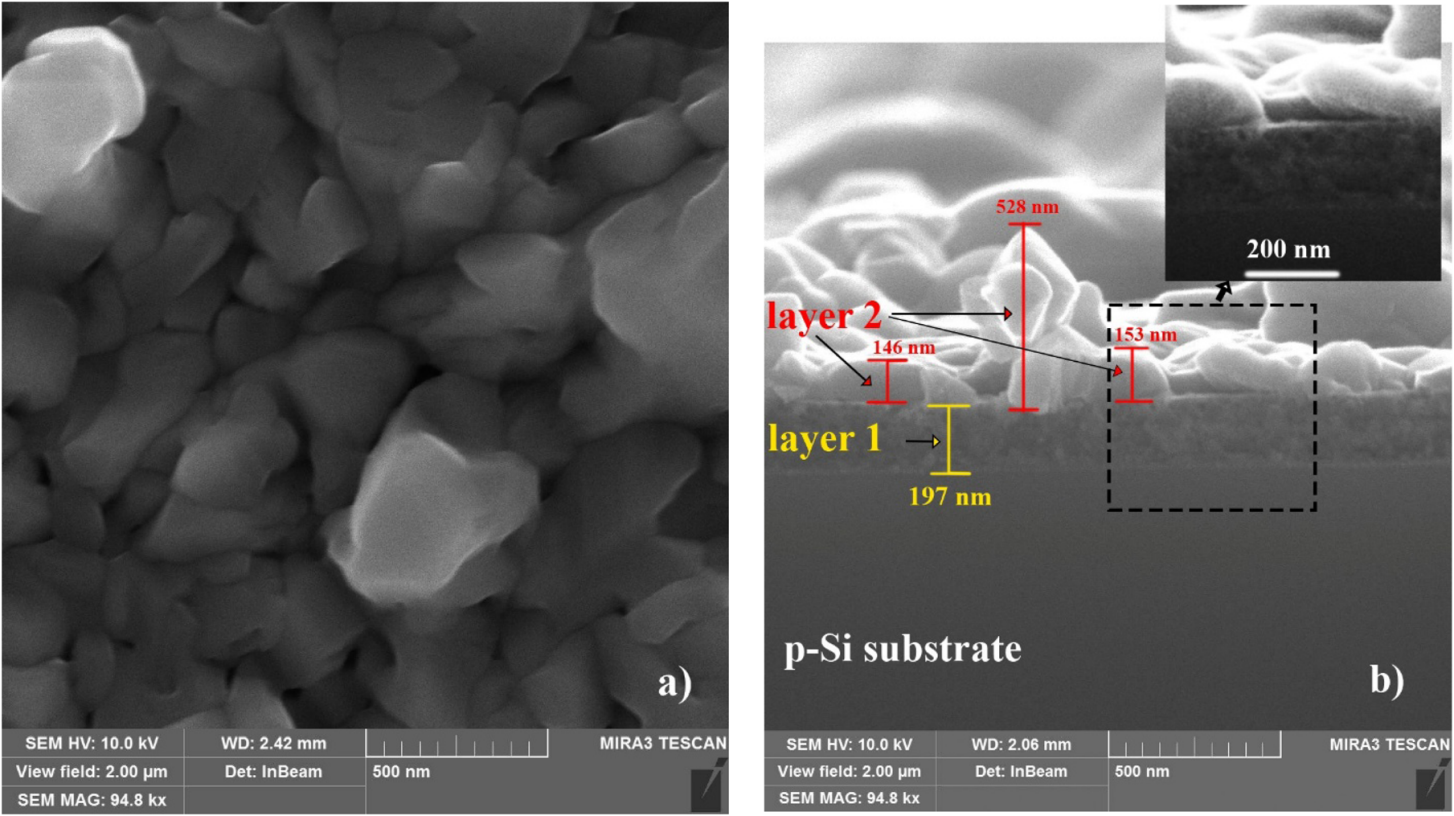
Surface (a) and cross-sectional (b) SEM images of the Cu–Si–O film on p-Si(100) after annealing at 600 °C (Ar, 30 min). The surface exhibits crystal-like features, and the cross-sectional image shows a pronounced two-layer film (layers 1 and 2).

Fig. 4 shows SEM images of the Cu–Si–O film annealed at 600 °C: surface (a) and cross-section (b). Annealing leads to the formation of a rocky surface texture with crystal-like structures ranging in size from 0.2 to 1.0 µm, where distinct edges and even facets begin to appear. These structures are separated by small granular gaps, indicating the possible crystallization of certain phases within the Cu–Si–O film (see Fig. 4a).

The cross-sectional view of the film shown in Fig. 4b reveals a decrease in the thickness of the bottom layer to *d*_1_ = 197 nm and a significant increase in the thickness of the top layer to *d*_2_ ≈ 150 nm, indicating annealing-driven redistribution of constituents from the bottom layer towards the top. Crystal-like formations with a height of up to ≈500 nm are formed on the surface of the top layer.

Thus, the as-deposited Cu–Si–O film exhibits a uniform granular structure with nanoscale contrast variations, indicating structural and/or compositional heterogeneity. Annealing at 400 °C leads to the formation of a bilayer structure, preserving the uniformity of the bottom layer, while rounded or elongated island-like structures appear on the surface. At *T*_ann_ = 600 °C, crystallization is suggested, resulting in a rough, rock-like texture and isolated pores. These observations indicate that the film structure and surface morphology are strongly dependent on the annealing temperature.

### 3.2. X-ray photoelectron spectra

The concentration of the main elements on the surface and the Cu : Si ratios are shown in Table 1. As seen in Table 1, copper tends to migrate to the surface at all annealing temperatures, forming a Cu-rich surface layer. After annealing at 600 °C, no silicon is detected on the surface. A small amount of carbon is also present on the film surface, with its concentration dropping to below 2% after ion etching. This may be due to contamination within the pores and at the grain boundaries of the crystallites.

**Table 1:** Surface elemental composition of Cu–Si–O films

*T*_ann_, °C	Cu : Si ratio	Cu, at%	Si, at%	O, at%
As-deposited	0.99 : 1	24.00	24.2	51.71
600	1 : 0	46.18	—	53.82

The absence of the Si signal after annealing at 600 °C does not necessarily indicate complete removal of silicon from the film. Rather, it shows that silicon is no longer detected within the shallow surface region probed by XPS. This behavior can be explained by thermally stimulated copper segregation toward the surface and the formation of a Cu-rich surface layer, which attenuates the photoelectron signal from the underlying Si-containing region. Due to the sampling depth for XPS analysis with photon energy 1486.6 eV being less than 10 nm, even a relatively thin copper-containing overlayer can strongly suppress or completely mask the Si 2p signal.

**Fig. 5:**
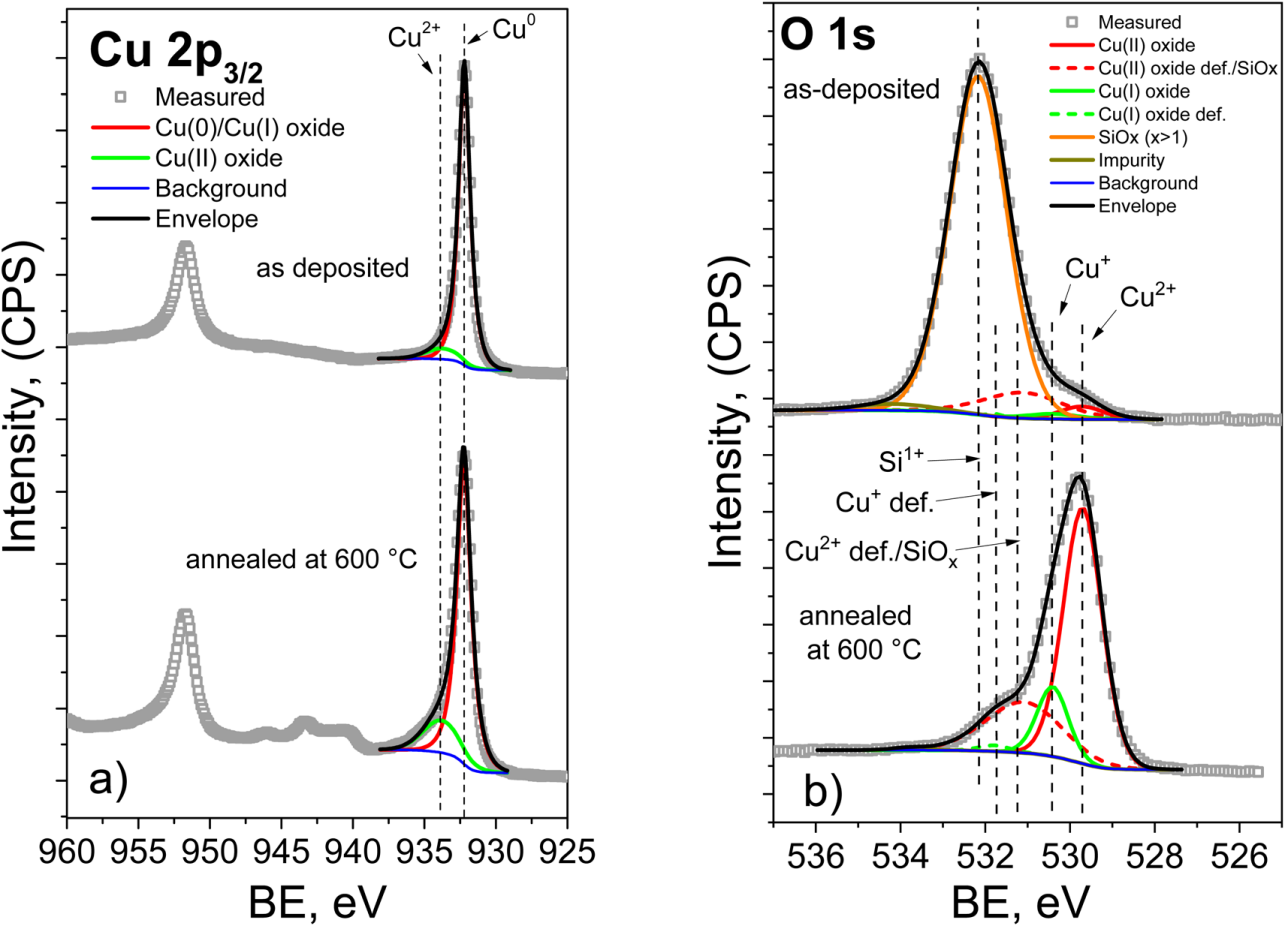
High-resolution XPS spectra of the Cu–Si–O film: (a) Cu 2p and (b) O 1s. In both panels, the upper spectrum corresponds to the as-deposited film, whereas the lower spectrum corresponds to the film annealed at 600 °C.

Fig. 5a shows the high-resolution spectra of the Cu 2p_3/2_ and Cu 2p_1/2_ peaks, as well as the Cu 2p_3/2_ peak deconvolution. The peak fitting was carried out using a Shirley background and a Gaussian–Lorentzian peak shape.^39^ For direct comparison, the spectra of the as-deposited and 600 °C-annealed films are presented within the same figure format. This makes it possible to clearly trace the annealing-induced evolution of the Cu chemical states and the corresponding spectral changes. As can be seen from the graph, the initial sample and the sample annealed at 600 °C exhibit the main Cu 2p^3/2^ feature at about 932.4 ± 0.1 eV. The Cu 2p^3/2^ peak fitting shows that copper present in two states: the overlapping peak from Cu^0^ and/or Cu^1+^ species, and Cu^2+^, which is identified from the fitted oxide component and, in particular, from the shake-up satellite structure.^32,39,40^ Annealing leads to the formation of a larger amount of the Cu^2+^ oxide phase, from ≈8 at% to ≈17 at%. This is also confirmed by the enhanced intensity of the shake-up peaks.

It should be noted that after annealing the most significant spectral change is not a large displacement of the main Cu 2p maximum, but rather a change in the line shape and in the relative contribution of the fitted components. This is expected, because the Cu^0^/Cu^+^ phase is dominant in both samples. Therefore, the annealing-induced chemical transformation is more reliably reflected by the increased Cu^2+^ contribution and stronger satellite intensity than by the position of the main maximum alone.

The deconvolution of the O 1s spectra (Fig. 5b) revealed peaks corresponding to oxygen in Cu_2_O and CuO within the crystal lattice (530.2 ± 0.1 eV and 529.7 ± 0.1 eV), as well as “defective” oxygen states (531.6 ± 0.1 eV and 531.0 ± 0.1 eV).^39^ In the initial sample, a peak at 532.2 ± 0.1 eV corresponds to oxygen in silicon oxide within the SiO_*x*_ structure. An increase in the area of the defective CuO oxygen peak relative to lattice oxygen may be associated with surface contamination and/or oxygen from silicon oxide with a lower oxidation state.

After annealing at 600 °C, the O 1s spectrum changes markedly: the SiO_*x*_ related contribution at higher binding energy disappears, while the contributions associated with copper oxides become dominant. At the same time, the overall O 1s envelope shifts toward lower binding energy. This shift reflects a change in the oxygen bonding environment, from a mixed Si–O/Cu–O surface composition in the as-deposited film to a surface layer dominated mainly by Cu–O bonds after annealing. Thus, the changes in the O 1s spectra are consistent with copper enrichment of the outermost surface and with the attenuation of the silicon-related XPS signal. In the spectra of the films annealed at 600 °C, only the signals associated with copper oxides and surface contamination are observed.

**Fig. 6:**
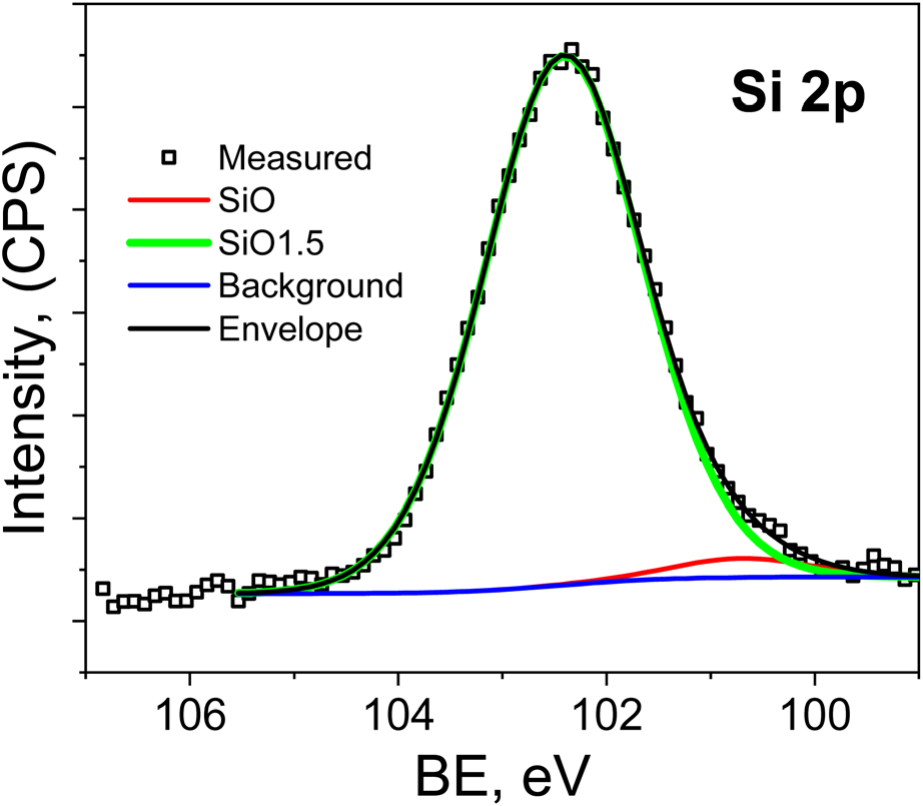
Si 2p high resolution spectra of as deposited Cu–Si–O films.

The analysis of the Si 2p spectra (Fig. 6) shows that the initial sample contains peaks at 102.4 ± 0.1 eV and 100.7 ± 0.1 eV. These correspond to Si–O bonds in SiO_*x*_ with *x* = 1.5 (Si^3+^) and *x* < 1 (Si^1+^), respectively.^41^ For the film annealed at 600 °C, no Si 2p signal is observed. In combination with the surface compositional data in Table 1 and the changes in the Cu 2p and O 1s spectra, this indicates that annealing causes strong copper enrichment of the near-surface region probed by XPS. As a result, the silicon-related signal becomes strongly attenuated and falls below the detection limit of the method.

Thus, XPS analysis confirms that annealing at 600 °C results in substantial chemical and compositional restructuring of the film surface. The surface becomes enriched in copper, the contribution of the CuO phase increases, the O 1s spectrum shifts toward copper-oxide-related states, and the Si signal disappears from the XPS-detectable region due to the surface sensitivity and limited probing depth of the method.

### 3.3. SIMS

**Fig. 7:**
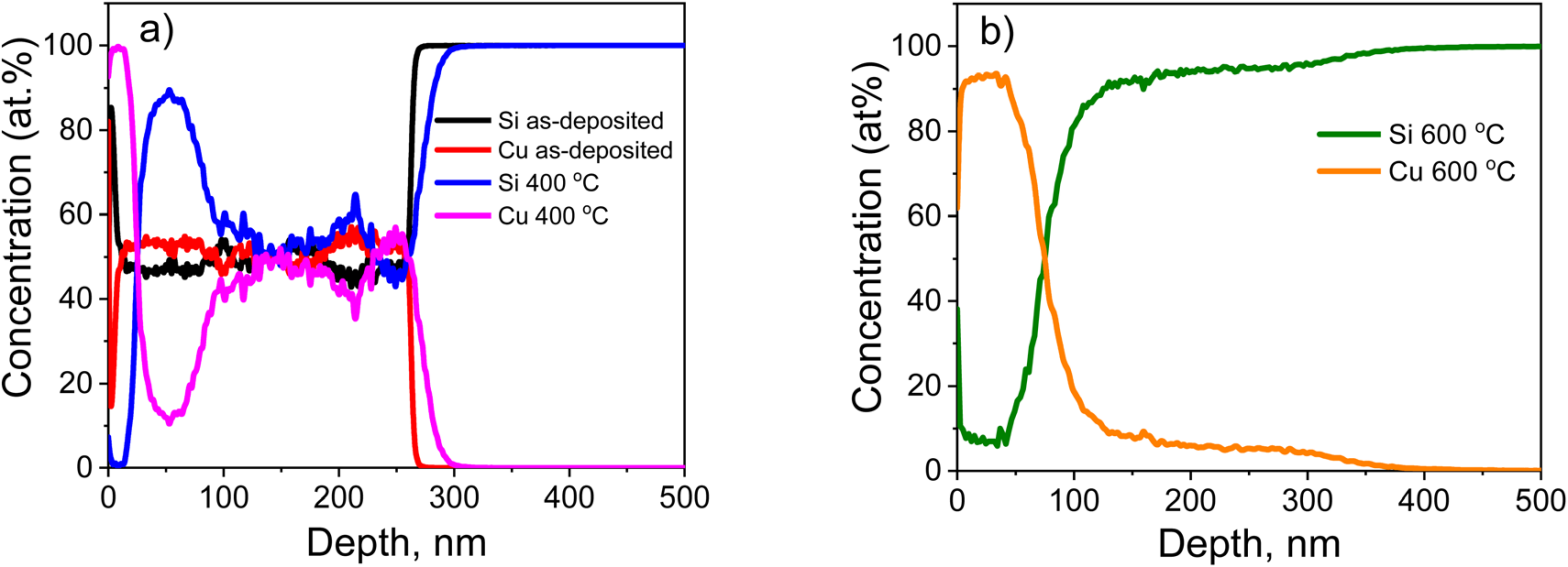
Normalized SIMS profiles of the concentration distribution of Si and Cu impurities along the depth of the Cu–Si–O film on the surface of the silicon substrate (p-Si): (a) before and after annealing at 400 °C; (b) after annealing at 600 °C.

Fig. 7a shows the normalized SIMS profiles of Si and Cu impurity distribution in the Cu–Si–O film on the silicon substrate before and after annealing at 400 °C. The vertical axis represents the relative values of *N*_Cu_/(*N*_Cu_ + *N*_Si_) × 100% (red and pink curves in the left figure), as well as *N*_Si_/(*N*_Cu_ + *N*_Si_) × 100% (black and blue curves in the left figure; olive and gray curves in the right figure). Here, *N*_Cu_ and *N*_Si_ denote the number of Cu and Si atoms (ions) per unit volume, respectively. This normalization of the signals allows tracking changes in the Cu-to-Si ratio in the fabricated structures after thermal treatments. A copper-implanted silicon sample was used as the reference sample.

For the as-deposited Cu–Si–O film (without annealing), an almost uniform distribution of Si and Cu atoms is observed along the depth. After annealing at 400 °C, a redistribution of Cu occurs throughout the film thickness: its concentration increases to a maximum in the near-surface layer (*d* < 15 nm). With increasing depth, the Cu concentration decreases, reaching a minimum at approximately *d* ≈ 50 nm, and then rises again to values close to those of the as-deposited sample. The Si distribution after annealing shows a mirror-like behavior relative to Cu. Segregation of Cu and Si is also observed. On the surface (*d* < 15 nm), a copper-rich layer without silicon is formed. Below this, in the region of 35 nm < *d* < 70 nm, a layer is observed with a Cu/Si ratio of ≈ 90/10. In the range of 100–270 nm, the Cu/Si ratio remains nearly constant. Additionally, a part of the copper atoms diffuses deeper into the silicon substrate.

Fig. 7b presents the normalized SIMS profiles of Si and Cu atoms distribution in the Cu–Si–O film on the silicon substrate after annealing at 600 °C. For the film annealed at 600 °C, the thickness of the near-surface Cu layer increases to *d* = 42 nm, with the Si content in this Cu-rich layer reaching about 5%. In the thickness range of 40 nm < *d* < 125 nm, a transition layer is located where the Cu-to-Si ratio gradually changes. In the range of 125 nm < *d* < 300 nm, a Si-rich layer is formed, with an approximate *N*_Cu_/*N*_Si_ ratio of ≈ 5/95.

Thus, after annealing at 600 °C, the film becomes strongly compositionally stratified along the depth and can be described as a bilayer-like heterogeneous Cu–Si–O film, with a Cu-rich upper region and a Si-rich lower region. This transformation indicates that annealing drives thermally activated phase separation and elemental redistribution within the film.

Based on the results of the SIMS method, it can be concluded that increasing the annealing temperature to 600 °C leads to a rise in the concentration of Cu atoms in the near-surface region of the Cu–Si–O film. At the same time, the overall depth distribution changes from nearly uniform in the as-deposited state to strongly stratified after high-temperature annealing.

**Fig. 8:**
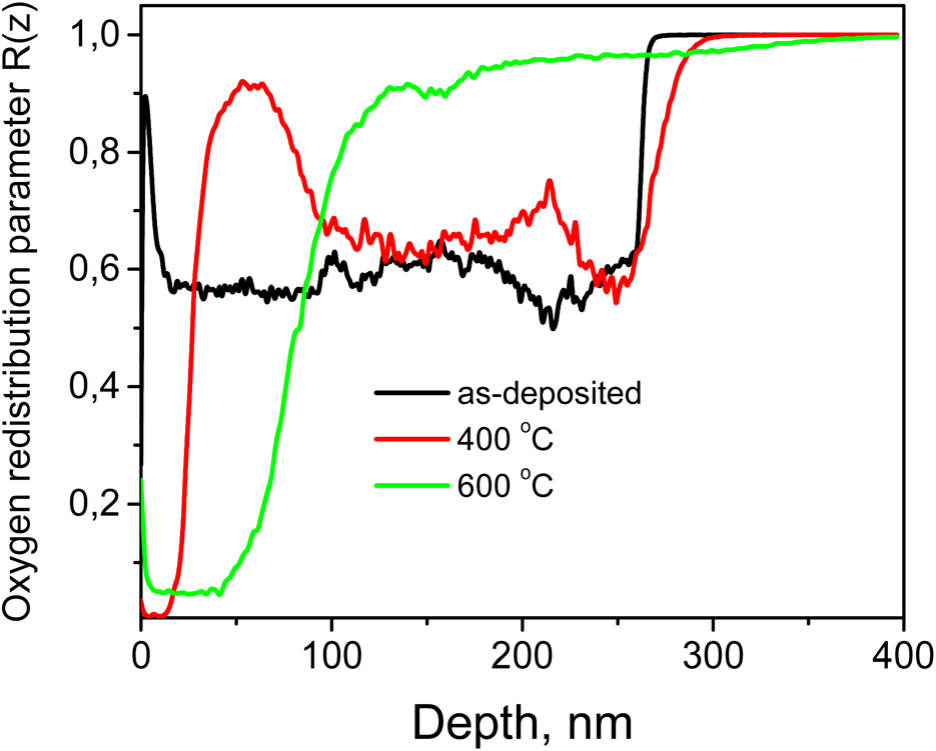
Depth profiles of the oxygen redistribution parameter for the Cu–Si–O film deposited on a silicon substrate in the as-deposited state and after annealing at 400 °C and 600 °C.

Fig. 8 shows the depth-dependent profiles of the oxygen redistribution parameter *R*(*z*) for the Cu–Si–O film deposited on a silicon substrate in the as-deposited state and after annealing at 400 °C, and 600 °C. The oxygen redistribution parameter was defined as the ratio of the total intensity of oxygen associated with Si–O related species to the total intensity of oxygen associated with both Si–O and Cu–O species: *R*(*z*) = *I*_Si–O_(*z*)/(*I*_Si–O_(*z*) + *I*_Cu–O_(*z*)).

This parameter does not represent the absolute oxygen concentration. Under dynamic SIMS conditions with an O_2_^+^ primary ion beam, the atomic O^+^ signal is strongly affected by ionization probability and matrix effects. Instead, *R*(*z*) reflects the relative partitioning of chemically bound oxygen between Si–O and Cu–O bonding configurations. The numerator accounts for oxygen associated with silicon, whereas the denominator represents the total amount of oxygen chemically incorporated in oxide-related species within the analyzed volume. Normalization to the total oxide signal minimizes matrix effects inherent to positive secondary ion detection and ensures that the observed variations primarily reflect chemical redistribution rather than changes in total ion yield.

As shown in Fig. 8, the as-deposited sample exhibits a relatively high but weakly depth-dependent *R*(*z*) distribution, indicating that a significant fraction of oxygen is already associated with silicon oxide species. This behavior is consistent with the incorporation of oxygen into both Si–O and Cu–O related environments during deposition. Higher *R*(*z*) values are observed in silicon-rich regions, while lower values are characteristic of copper-containing layers, indicating different oxygen incorporation/stabilization behavior in the Cu-rich and Si-rich regions.

After annealing at 400 °C, moderate but spatially non-uniform changes in *R*(*z*) are observed. In particular, local maxima of *R*(*z*) appear within silicon-rich regions at depths of approximately 50 nm and 200 nm. These features indicate the onset of oxygen accumulation in silicon-rich regions, likely driven by defect-assisted diffusion toward energetically favorable Si–O bonding configurations near the Cu/Si interfaces. At this temperature, oxygen mobility is sufficient for local redistribution and trapping in defect-rich regions but remains limited, resulting in a non-uniform stabilization of oxygen within the silicon-rich part of the film.

A pronounced redistribution occurs after annealing at 600 °C, where *R*(*z*) increases systematically in silicon-rich regions and decreases in copper-containing layers, indicating enhanced oxygen diffusion and interlayer transfer. In the near-surface Cu-rich region, the relatively low *R*(*z*) values indicate that oxygen is predominantly associated with Cu–O bonding. In contrast, in the deeper Si-rich region, *R*(*z*) increases strongly and approaches unity, showing that oxygen becomes predominantly associated with silicon oxide species. These results demonstrate that, although annealing was performed in an Ar atmosphere, oxygen already incorporated into the film during deposition undergoes substantial internal redistribution during annealing. Therefore, the formation of the CuO-rich upper region at 600 °C is governed not by an external oxygen supply, but by thermally activated redistribution of oxygen already present in the Cu–Si–O film.

Thus, the combined analysis of Cu, Si, and *R*(*z*) depth profiles shows that annealing causes not only Cu segregation toward the surface, but also concurrent redistribution of chemically bound oxygen between Cu–O and Si–O rich regions. This process drives the transformation of the initially nearly uniform Cu–Si–O film into a depth-stratified bilayer-like heterogeneous film.

The pronounced compositional stratification observed after annealing at 600 °C can be attributed to thermally activated diffusion and chemically driven redistribution processes in the Cu–Si–O film. Upon annealing, Cu atoms exhibit a strong tendency to migrate toward the surface, whereas Si remains preferentially stabilized in the oxide-rich deeper region of the film. Such behavior is consistent with diffusion-driven phase separation reported for related co-sputtered metal–oxide systems, where differences in elemental mobility and in the thermodynamic stability of oxide phases govern depth-dependent segregation. In particular, Cu out-diffusion accompanied by structural rearrangement of the SiO_*x*_-based matrix and formation of segregated regions has been reported for Cu–SiO_2_-derived films during annealing.^32^ A similar trend was also demonstrated for co-sputtered Cu(Ti) films, in which annealing induced phase separation, enrichment of Cu near the surface, and segregation of the more oxygen-affine component toward the interface region.^42^ In the present case, the redistribution of oxygen within the film additionally contributes to the stabilization of Cu–O bonds in the near-surface region and Si–O bonds in the deeper layer. As a consequence, the initially nearly uniform Cu–Si–O film evolves into a depth-stratified bilayer-like structure after high-temperature annealing.

In summary, SIMS analysis shows that the as-deposited Cu–Si–O film is characterized by an almost uniform depth distribution of Cu and Si, whereas annealing induces progressive elemental segregation. After annealing at 600 °C, the film becomes strongly stratified and bilayer-like, with a Cu-rich upper region and a Si-rich lower region. The *R*(*z*) profiles further demonstrate that oxygen, initially incorporated during deposition, is internally redistributed during annealing in Ar between Cu–O and Si–O related environments.

### 3.4. X-ray diffraction method

**Fig. 9:**
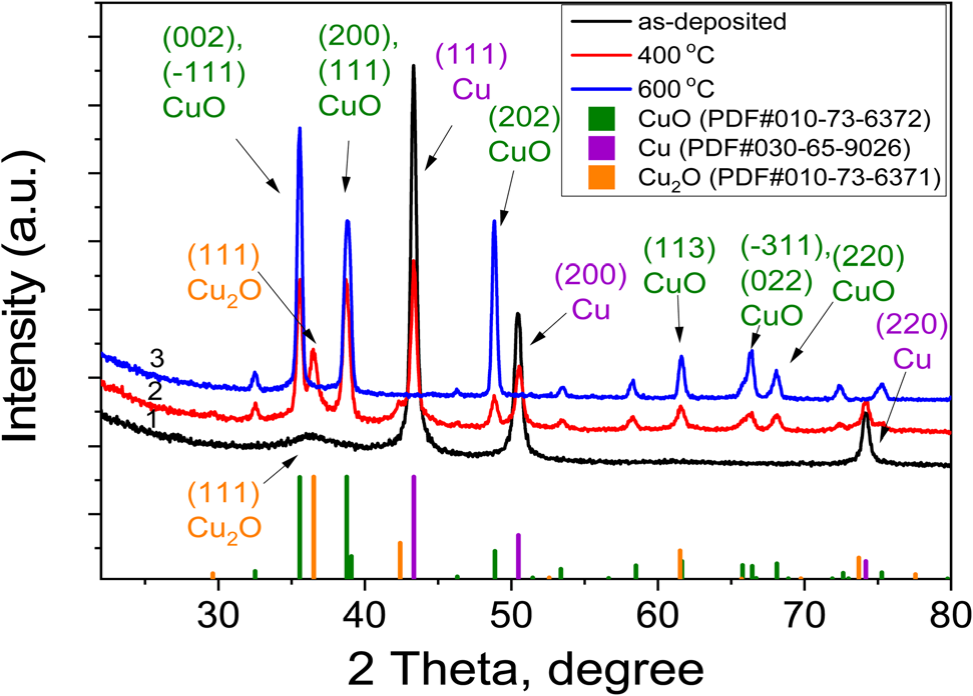
XRD patterns of the Cu–Si–O film deposited on p-Si(100) substrates, measured in grazing-incidence geometry, before and after annealing at 400 and 600 °C.

Quantitative phase composition analysis and refinement of lattice parameters were carried out using full-profile analysis of the diffractograms (Rietveld method). In the diffractogram of the as-deposited sample (Fig. 9, curve 1), strong reflections are observed at 43.34°, 50.47°, and 74.17°, corresponding to the (111), (200), and (220) reflections of the cubic phase of copper (PDF no. 030-65-9026). A weak peak at 36.24° most likely indicates the (111) reflection of the cubic Cu_2_O phase (PDF no. 010-73-6371), suggesting that copper oxidation begins during deposition.

After annealing the as-deposited sample at 400 °C, additional peaks of the monoclinic CuO phase (PDF no. 010-73-6372) and cubic Cu_2_O phase appear, while the intensity of the Cu reflections decreases (Fig. 9, curve 2). The phase composition includes 55.6% CuO, 15.8% Cu_2_O, and 28.6% Cu. Following annealing at 600 °C, only reflections of CuO remain (Fig. 9, curve 3), indicating that the oxidation of copper is nearly complete at this temperature.

The diffractograms of the samples annealed at 600 °C show a set of reflections (Fig. 9, curve 3), which correspond to the monoclinic CuO phase and do not contain any other crystalline phases. Using the Williamson–Hall method,^43–47^ the average size of the coherent scattering regions (*D*, nm) and the average level of microstrain (*ε*, %) in the present phases were determined (see Table 2). The Williamson–Hall analysis was performed separately for each crystalline phase using the diffraction peaks assigned to that phase.

**Table 2:** Structural parameters (*D*, *ε*) and relative concentrations of the crystalline phases in the initial film and after annealing at 400 and 600 °C

*T*_ann_, °C	Cu			Cu_2_O			CuO		
	*D*, nm	*ε*, %	Phase conc., %	*D*, nm	*ε*, %	Phase conc., %	*D*, nm	*ε*, %	Phase conc., %
As-deposited	15.0	0.01	98.6	—	—	1.4	—	—	—
400	14.1	0.12	28.6	45.2	1.14	15.8	65.3	0.83	55.6
600	—	—	—	—	—	—	36.1	0.14	100

The latter parameter to some extent characterizes the degree of crystalline perfection of the materials, since point defects and their accumulation lead to the occurrence of local deformations in the crystallites, which contribute to the broadening of the reflections. It should be noted that this value is averaged and includes both tensile and compressive microstrains. The concentrations of the crystalline phases determined by the Rietveld method are listed in Table 2.

The absence of diffraction peaks associated with Si or SiO_2_ in the XRD patterns is due to the structural state of the silicon-containing phase in the investigated films. In the as-deposited film, as well as in the samples annealed at 400 and 600 °C, silicon is present predominantly in an amorphous state in the form of SiO_*x*_ and/or amorphous Si nanoclusters, which do not form long-range crystalline order and therefore are not detected by X-ray diffraction. It is known that the formation of crystalline Si nanoparticles in a dielectric SiO_*x*_ matrix occurs only at higher annealing temperatures (typically ≥ 800–900 °C),^20^ when phase separation and crystallization of excess silicon take place.

Thus, in the as-deposited film, the crystalline Cu phase is predominant (98.6%), with a small amount of the Cu_2_O phase (1.4%). Annealing the film at 400 °C indicates the beginning of the oxidation process of crystalline Cu, leading to the formation of the CuO phase (55.6%) and further oxidation of the Cu_2_O phase (15.8%). At *T*_ann_ = 600 °C, the crystalline Cu and Cu_2_O phases disappear completely, resulting in the formation of a single-phase CuO composition (100%).

### 3.5. Raman spectroscopy

**Fig. 10:**
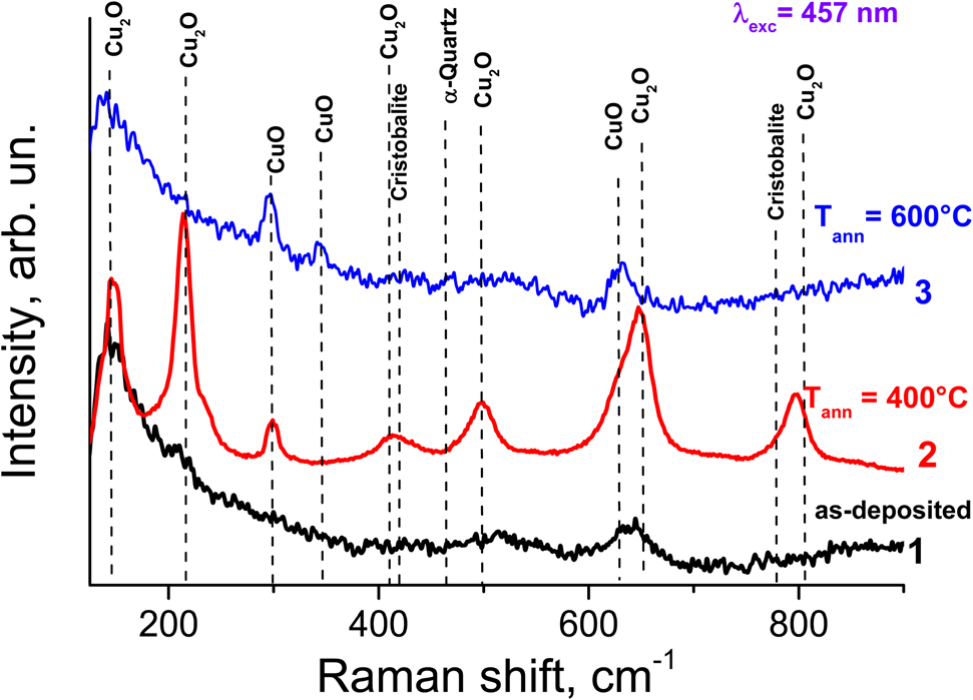
Raman spectra of Cu–Si–O films formed on Si substrates: directly after deposition (1) and after annealing in an argon atmosphere for 30 minutes at 400 °C (2) and 600 °C (3).

Fig. 10 shows the Raman spectra of Cu–Si–O films formed on Si substrates: directly after deposition (1) and after thermal annealing in an argon atmosphere for 30 minutes at temperatures of 400 (2) and 600 (3) °C. After deposition, two broad spectral features appear with maxima at 500 and 640 cm^−1^. The first feature may correspond to amorphous silicon, while the second is likely related to Cu–O vibrational bonds.

Annealing the film at 400 °C leads to the formation of crystalline CuO and Cu_2_O phases. As is well known, CuO has a monoclinic structure and belongs to the *С*2/*c* space group. According to group theory analysis, 12 phonon modes can appear in the vibrational spectra of CuO: *Γ* = 4A_u_ + 5B_u_ + A_g_ + 2B_g_. Among them, three modes (A_u_ and 2B_u_) are acoustic, and the remaining nine are optical, and six of them (3A_u_ and 3B_u_) are IR-active, while the remaining three (A_g_ and 2B_g_) are Raman-active. In our experimental Raman spectra, they appear at 296, 346, and 630 cm^−1^ and correspond to the A_g_, B_g_, and B_g_ vibrational modes, respectively.^48^

As for Cu_2_O crystals, their unit cell contains two formula units, and according to group theory analysis, fifteen optical and three acoustic vibrational modes can appear in the spectra (*Γ*_Cu_2_O_ = A_2u_ + E_u_ + 3T_1u_ + T_2u_ + T_2g_). Among these vibrational modes, only T_2g_ is Raman-active. In our case, this corresponds to the band at 515 cm^−1^. However, as seen in Fig. 10 (spectrum 2), the experimental Raman spectrum shows six bands associated with Cu_2_O at 146, 217, 310, 417, 520, 636, and 810 cm^−1^. The appearance of these modes in the spectrum is the result of selection rule violations, caused by defects present in the films and/or possible mechanical stresses within the film structure.

It should be noted that the intensity of the bands associated with Cu_2_O is significantly higher than that of CuO. However, it cannot be definitively concluded that the amount of CuO in the film is lower than that of Cu_2_O, since the efficiency of Raman scattering may be influenced by the realization of resonance Raman conditions during spectrum acquisition. Indeed, the bandgap energies of CuO and Cu_2_O are 1.4 eV and 2.38 eV, respectively.^49^ Therefore, excitation of Raman spectra with laser radiation of 2.7 eV (457 nm) will be more effective for Cu_2_O, especially in the case of quantum-sized crystals, for which the bandgap increases compared to bulk Cu_2_O. The dominance of Cu_2_O and CuO features is further enhanced by the resonance Raman effect, as the excitation energy (2.71 eV) is close to the electronic transitions of these semiconductor oxides, while SiO_*x*_ remains transparent and non-resonant. Furthermore, the absence of distinct SiO_*x*_ bands is attributed to the low Raman scattering cross-section of the amorphous oxide matrix compared to the crystalline copper oxide phases.

Annealing the film at 600 °C leads to the formation of only the CuO phase (see spectrum 3, Fig. 10). Such a drastic change in the Raman spectrum compared to spectrum 2 may indicate that during annealing at 600 °C, oxygen atoms are released and bond with copper atoms. This is supported by the fact that the Gibbs free energy for the CuO phase is −45.89 kJ per (mol atom), which is significantly lower than the corresponding value for Cu_2_O (−39.22 kJ per (mol atom)). Therefore, in our case, CuO is preferentially formed. Similar results were reported in ref. 50 for Cu–O films annealed at 600 °C.

Thus, Raman spectroscopy was used to study the phase-structural transformations in initial Cu–Si–O films at annealing temperatures ranging from 400 °C to 600 °C. It was shown that in films annealed at 400 °C, both Cu_2_O and CuO phases are present. In contrast, at 600 °C, only the CuO phase forms, since its Gibbs free energy is lower than that of Cu_2_O. The obtained results are in good agreement with the data acquired by XRD.

### 3.6. Influence of the annealing temperature on the IR transmission spectra of Cu–Si–O films

**Fig. 11:**
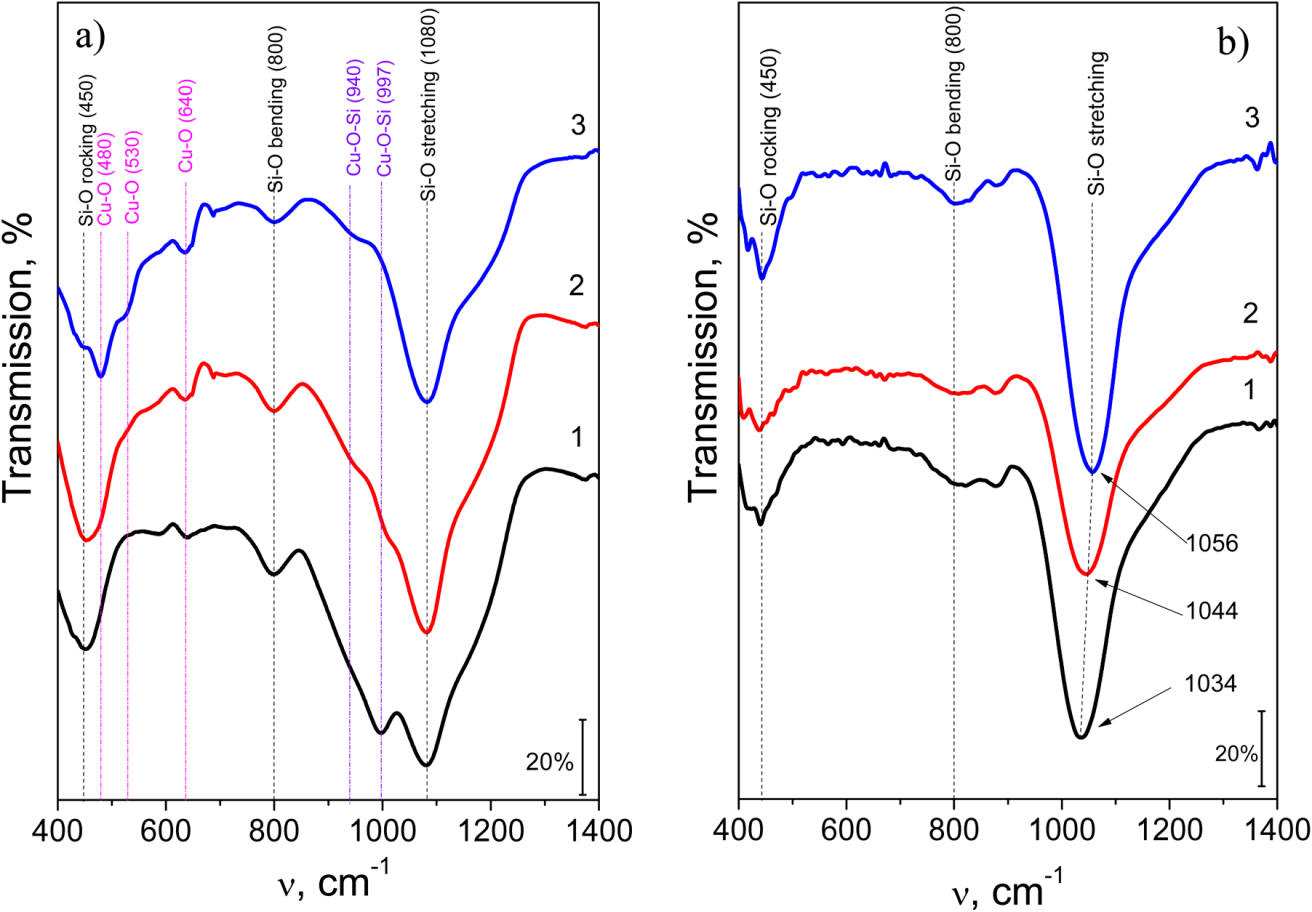
IR absorption spectra of Cu–Si–O films (a) and SiO_*x*_ films (b) before (curve 1) and after annealing at temperatures of 400 (2), and 600 (3) °C. Spectral positions of Cu–O and Si–O vibrational modes are indicated for clarity.

The evolution of the Cu–Si–O film structure with increasing annealing temperature was studied using infrared (IR) transmission spectroscopy. Fig. 11a shows the transmission spectra of the Cu–Si–O film recorded under normal incidence of the excitation light. To identify the contribution of vibrational modes associated with Cu–O and Si–O bonds, these spectra were compared with the IR transmission spectra of SiO_*x*_ films of similar thickness that were annealed under the same conditions (Fig. 11b).

It is known that Si–O vibrational modes appear in the range of 400–1300 cm^−1^. Under normal incidence of IR excitation light, the presence of Si–O bending, rocking, and stretching modes can be observed near 450–490 cm^−1^, 780–810 cm^−1^, and 1040–1080 cm^−1^, respectively (Fig. 11a), which correspond to the vibrations of oxygen atoms relative to silicon atoms.^51^

As for Cu–O modes, they are typically observed in the range of 300–700 cm^−1^, in particular at 610 cm^−1^ (TO) and 650 cm^−1^ (LO) for Cu_2_O (cuprous oxide).^52^ Vibrational modes for CuO (cupric oxide), which has a monoclinic structure, appear at 480 cm^−1^ and 530 cm^−1^ (TO), and 580 cm^−1^ (LO).^51^ In other studies, it is shown that for Cu_2_O and CuO the modes at 620 cm^−1^ and at 510 cm^−1^, respectively, are active.^53^ The spectral position of these modes may shift to 640 cm^−1^ and 520–560 cm^−1^, respectively, with increasing oxide film thickness.^52^ IR transmission spectra for CuO particles coated with a SiO_2_ shell show slightly different Cu–O vibrational mode positions – at 468 cm^−1^, 554 cm^−1^, and 877 cm^−1^,^54,55^ indicating that the shape and composition of the copper oxide affect the position of the IR bands.

The IR transmission spectra of Cu–Si–O films show several bands that can be clearly attributed to Cu–O and Si–O vibrations. Notably, the spectral positions of the Si–O “bending”, “rocking”, and “stretching” modes^51^ peaked at 458 cm^−1^, 800 cm^−1^, and 1080 cm^−1^, respectively, remain unchanged regardless of annealing temperature (Fig. 11a, curves 2,3). The appearance of intense Si–O–Si mode at 1080 cm^−1^ can be explained by the formation of asymmetric stretching vibration of the bridging oxygen specific for high-quality stoichiometric SiO_2_. This statement is supported by the evolution of IR spectra of a SiO_*x*_ film with annealing (Fig. 11b). Usually, in amorphous substoichiometric SiO_*x*_, this peak appears at lower frequency, observed here at 1034 cm^−1^. Upon annealing, its progressive shift to 1056 cm^−1^ (Fig. 11b) is caused by the relaxation of strained Si–O bonds toward their equilibrium tetrahedral geometry with a bridging bond angle of about 144°. This relaxation increases also the force constant favoring the shift of the Si–O–Si peak position to high frequency range observed in SiO_2_. Saying in advance, the Si–O–Si peak at about 1080 cm^−1^ (Fig. 11a) can originate from the phase separation within Cu–Si–O network accompanied by the decrease of the magnitude of the Cu–O–Si peak at 1000 cm^−1^ and formation of relaxed Si–O–Si bonds specific for high-quality SiO_2_ (Fig. 11a).

Indeed, examination of the Cu–O and Si–O vibrational band positions suggests significant overlap between the bands. The band in the 440–460 cm^−1^ range is a superposition of the Si–O–Si “bending” mode and a Cu–O mode, while the band in the 900–1030 cm^−1^ region appears as a broadening of the Si–O–Si “stretching” mode due to the emergence of a maximum at 997–1000 cm^−1^, which is attributed to the formation of Cu–O–Si bonds during the film deposition process (Fig. 11a, curves 1–3). A similar effect, where an element of a different nature than silicon is incorporated into Si–O–Si bonds, leading to a shift of the “stretching” Si–O–Si band to lower frequencies, has been observed in SiO_2_ films doped with hafnium and/or rare-earth elements.^56,57^ A comparable effect related to the formation of Ti–O–Si bonds was demonstrated in ref. 58.

In Cu–Si–O films annealed at 400 °C, additional modes are observed at 485, 520, 640, and 940 cm^−1^ (shoulder), while the contribution of the peak at 997–1000 cm^−1^ decreases (Fig. 11a, curve 2). As *T*_ann_ increases to 600 °C, new modes appear (at 430 cm^−1^ as well as at 655 and 680 cm^−1^), along with a reduction in the contribution of the 940 cm^−1^ mode to the IR transmission spectrum (Fig. 11a, curve 3).

FTIR studies of Cu–Si–O films show that in the as-deposited film, the intensity of Si–O bands significantly exceeds that of Cu–O bands. However, since the copper and silicon contents are approximately equal, it can be assumed that copper may be present in the form of isolated atoms (Cu^0^) or copper clusters embedded in the silicon oxide matrix. As for the presence of Cu–O bonds, they may originate from a phase present within the bulk of the film or on the surface of copper clusters.

### 3.7. Tracking the transformations of Cu–Si–O films through analysis of absorption spectra in the near band and inter-band absorption regions

The aim of this section is to investigate the optical spectra of Cu–Si–O multiphase films in the electron absorption region to gain further insight into their properties.

#### 3.7.1 Qualitavite analysis of the *R*–*T* spectra

**Fig. 12:**
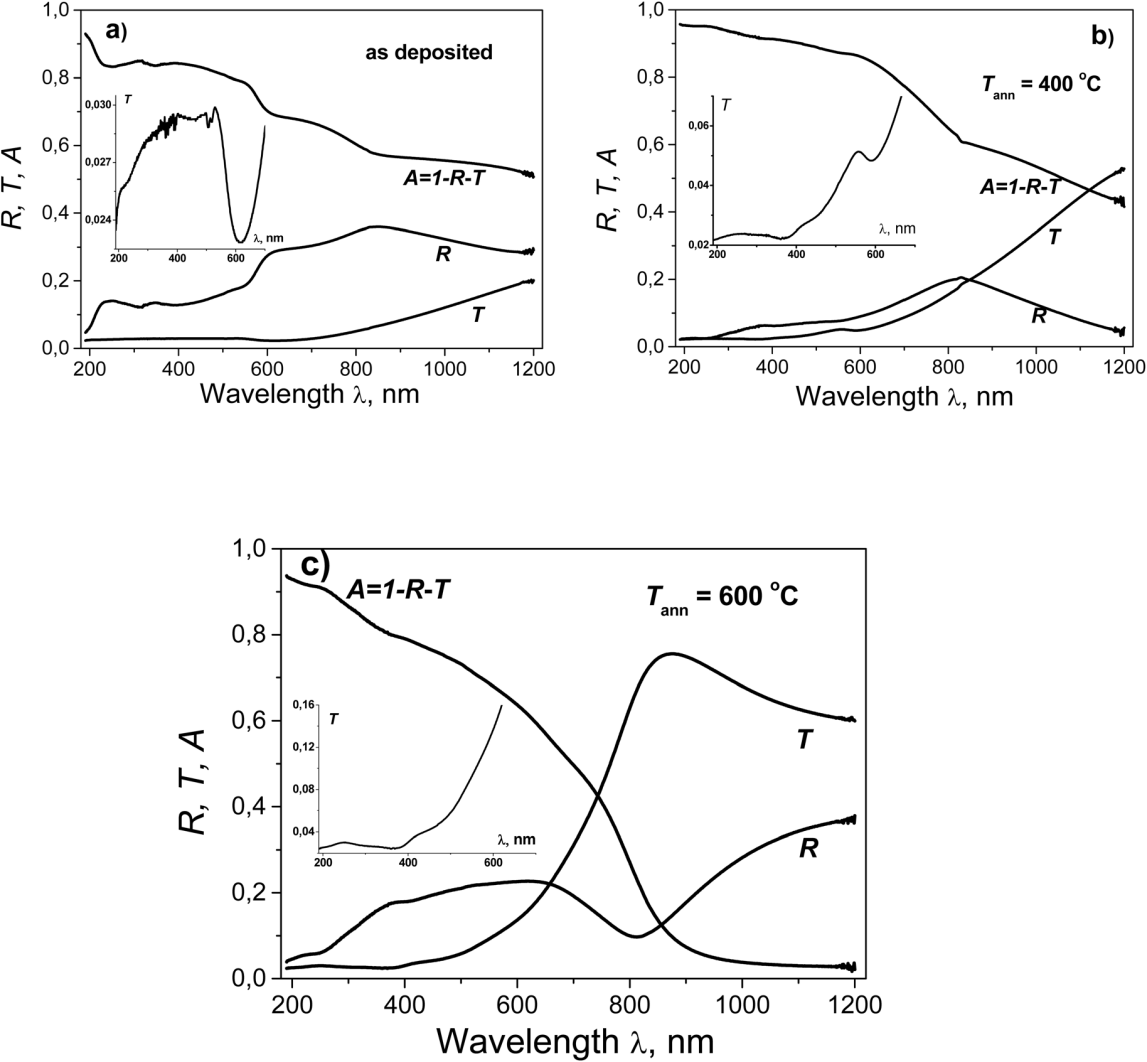
Reflectance, transmittance and absorptance spectra for the Cu–Si–O film: (a) as deposited; (b) after annealing at 400 °C; (c) after annealing at 600 °C.

‘Try graphics first’ is one of the basic scientific principles when analyzing data in many research areas.^59,60^ In our case, it is useful to first consider the spectral dependencies of *R*–*T* and try to extract as much information as possible from them. The measured *T*(*λ*) and *R*(*λ*) spectral plots, along with the absorptance spectra (*A* = 1 − *R* − *T*), are shown in Fig. 12 (it should be noted that the *A* contains not only absorption effects but also scattering effects of light through the structure and the various interfaces of our films).

The general appearance of the transmittance spectra is characterised by low transmittance in the UV region (less than 3% for all films) and an increase in transmittance as the wavelength increases. The higher the annealing temperature, the stronger this increase. As a result, at the long-wavelength edge of the spectrum, the film annealed at 600 °C becomes almost completely transparent (*A* ≈ 0.025). In the deposited film, a well-defined minimum *T* is observed in the region of ∼615 nm (*hν* ∼ 2.0 eV). This shifts to ∼590 nm (*hν* ∼ 2.1 eV) after annealing at 400 °C, disappearing completely after annealing at 600 °C. In the short-wavelength region, against the background of a generally weak change in *T*, a weakly pronounced minimum is observed at ∼450 nm (*hν* ∼ 2.75 eV). After annealing at 400 and 600 °C, this minimum transforms into an inflection point on the *T*(*λ*) curves. After annealing at 400 °C, a minimum appears on the *T*(*λ*) plot at ∼365 nm (*hν* ∼ 3.40 eV), which deepens after annealing at 600 °C.

Due to the films' very low transmittance in the short-wavelength part of the spectrum, some features of the absorption spectrum may be more clearly visible in the *R*(*λ*) spectra. The *R*(*λ*) spectrum of the initial film shows several absorption bands, which could be due to interband and/or resonance absorption. Due to the film's complex, heterogeneous (multi-component) structure, these bands appear in the *R*(*λ*) spectrum as maxima, shoulders, and inflection points. These absorption bands are visually manifested at ∼850 nm (*hν* ∼ 1.46 eV) as a maximum; at ∼635 nm (*hν* ∼ 1.95 eV) as a shoulder; at ∼345 nm (*hν* ∼ 3.6 eV) as a maximum; and at ∼240 nm (*hν* ∼ 5.15 eV) as a maximum. The overall appearance of the *R*(*λ*) spectrum of a film annealed at 400 °C resembles a smoothed spectrum of a sputtered film with reduced total reflection. This may be because the two-layer film formed during annealing is more ‘anti-reflective’ than the original film. At the same time, the amount of energy absorbed by the film (*A*) increased across most of the spectrum (*λ* < 950 nm), due in part to improved light penetration in it. The *R*(*λ*) spectrum shows a broad maximum at 830 nm (*hν* ∼ 1.5 eV) and weak maxima at 385 nm (*hν* ∼ 3.22 eV) and 220 nm (*hν* ∼ 5.65 eV), as well as a shoulder at 540 nm (*hν* ∼ 2.30 eV). The long-wavelength part of the *R*(*λ*) spectrum of the film annealed at 600 °C is primarily due to interference effects. In the region of strong absorption (low transmittance), shoulders are observed at ∼380 nm (*hν* ∼ 3.25 eV) and ∼230 nm (*hν* ∼ 5.4 eV).

The minimum *T* at *hν* ∼ 2.0 eV and the shoulder in the *R* spectrum at *hν* ∼ 1.95 eV in the as-deposited film may be due to the superposition of the interband absorption edge of the copper particles, their surface plasmon resonance absorption band, and the near-edge absorption band of the Cu_2_O phase due to direct forbidden transitions.^61–63^ The weaker manifestation of the features in the *T*(*λ*) and *R*(*λ*) spectra of this region after annealing at 400 °C correlates precisely with the decrease in signals from the copper subphase in the XRD spectrum. The position of the shoulder in the *R*(*λ*) spectrum at 520 nm (∼2.4 eV) is close to the energy position of the optical band gap for direct transitions in Cu_2_O^63^ as well as the energy position of the first interband absorption peak in copper.^61^ The maximum at ∼250 nm (∼4.95 eV) is close to the position of the second interband absorption peak in copper.^61^ The reduction in the intensity of these features after annealing at 400 °C and 600 °C is consistent with the reduction in the XRD signal from copper. The position of the *T*(*λ*) minimum at ∼3.4 eV, which is observed after annealing at 400 °C and is enhanced after annealing at 600 °C, coincides with the positions of the strongest peaks of the optical dielectric function *ε*(*hν*) of CuO and Cu_2_O.^64,65^

The very broad maximum in the reflectance spectrum of the as deposited film is apparently the result of several factors. These include an increase in the effective refractive and absorption indices of the multicomposite in this region, due to an increase in edge absorption in the non-metallic subphases of the film. This is compounded by absorption in copper nanoparticles and, possibly, a geometric factor (interference effect). The narrowing and weakening of this maximum and its short-wavelength shift to 830 nm after annealing at 400 °C can be explained by a decrease in the copper nanoparticle content and structural ordering of the non-metallic subphases, or an interference effect. After annealing at 600 °C, this effect becomes dominant in the long-wavelength part of the investigated spectral range.

#### 3.7.2 Quantitative analysis of the absorption coefficient spectra

Photometry allows the average values of optical constants of complex films to be obtained more easily than other optical methods, *e.g.*, ellipsometry, where this requires complex modeling.^66,67^ For this reason, spectra of the absorption coefficient measured by spectrophotometry allow more direct access to the band gap energies, especially in the presence of sub-gap absorption.^68^ The absorption coefficient *α* of the films was determined using the following formula:^69^1
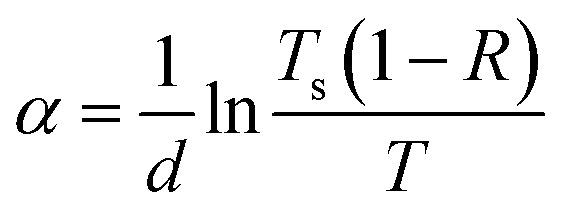
where *d* is the film thickness, *T*_s_ is the transmittance of the substrate without film, and *R* and *T* are the reflectance and transmittance of the film/substrate system, respectively.

**Fig. 13:**
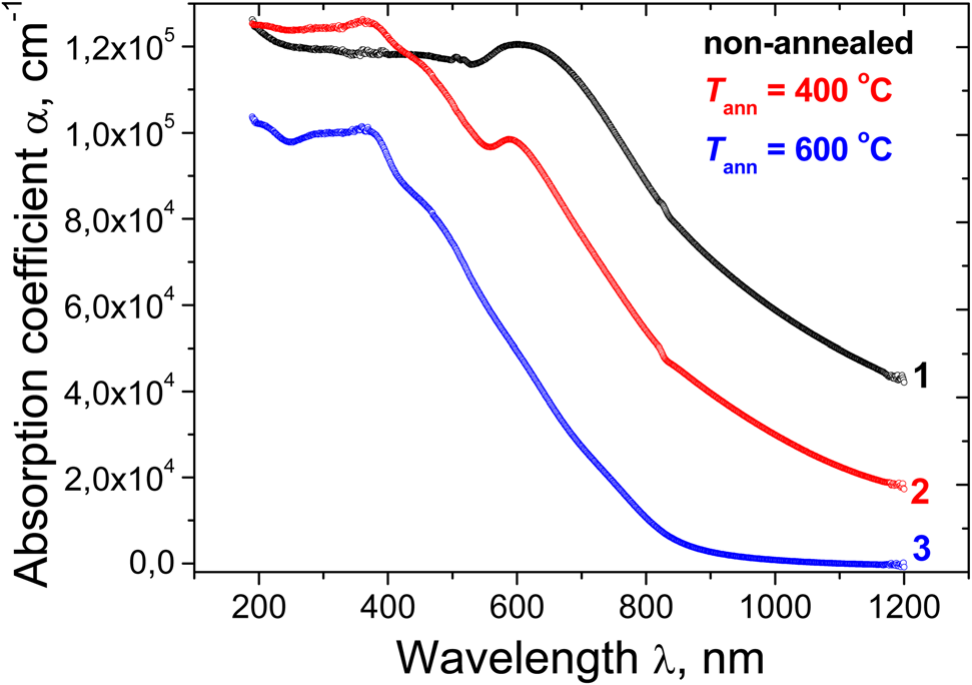
Spectral dependences of the absorption coefficient for the as-deposited Cu–Si–O film before (1) and after annealing at 400 (2), 600 °C (3).

The *α*(*λ*) spectra calculated on the basis of photometric measurements using eqn (1) are presented in Fig. 13. When moving toward shorter wavelengths, the growth rate of *α* first increases (fundamental absorption edge), after which a region of relatively small changes in *α* is observed (fundamental absorption region). The *α*(*λ*) dependence in the deposited film exhibits a maximum at *λ* ≈ 605 nm, obviously due to localized surface plasmon resonance (LSPR) in Cu nanoparticles. After annealing at 400 °C, this resonance peak shifts to shorter wavelengths and becomes less pronounced. After annealing at 600 °C, it is no longer observed. Both annealed films exhibit broad peaks at ∼365 nm (*hν* ≈ 3.4 eV) which is a distinct feature of crystalline Cu_2_O, Cu_4_O_3_ and CuO.^70^

In amorphous, disordered, heterogeneous, nanostructured semiconductor and dielectric materials, and semiconductor–dielectric nanocomposites there is usually a region of the absorption spectra generally termed the “Urbach region” where spectra *α*(*hν*) can be represented by the exponential form:^71,72^

2 *α* ∝ exp(*hν*/*E*_U_)

where *E*_U_ is called the Urbach tail energy. The value of *E*_U_ is equal to the inverse slope of the linear part of the ‘ln *α versus hν*’ plot. The absorption in this region is caused by the superposition of transitions from localized states in the valence band tail to non-localized states in the conduction band and transitions from non-localized states in the valence band to localized states in the conduction band tail. The photon energy at which a deviation from the Urbach law is observed can be used as empirical estimation of the electronic band gap *E*_g_ – the energy distance between the highest non-localized states in the valence band and the lowest non-localized states in the conduction band. The electronic band gap is typically derived from transport measurements. However, it is often very useful to estimate it from optical experiments.

The absorption spectrum in the region of interband transitions from non-localized states in the valence band to non-localized states in the conduction band can be approximated by the Tauc dependence:^73^

3 (*αhν*)^*m*^ ∝ (*hν* − *E*_0_)

where *E*_0_ is termed the optical band gap or the Tauc band gap. In eqn (3), *m* depends on the electron transitions exhibited by the material: *m* = 1/2, 2, 1/3, 2/3 for indirect allowed, direct allowed, indirect forbidden and direct forbidden transitions, respectively. The values of *E*_g_ and *E*_0_ should ideally be equal in magnitude, by definition. *E*_0_ is therefore regarded as an empirical quantity^74^ used to estimate the electronic band gap *E*_g_. However, as a general rule, the value of *E*_0_ obtained from the experimental results using the approximation (eqn (3)) is smaller than the true value of *E*_g_. The reason is the presence of structural and compositional disorder (including nanoheterogeneity), which gives rise to the Urbach tail effect. The larger the *E*_U_, the greater the difference between the true value of *E*_g_ and the value of *E*_o_ obtained by approximating the experimental curve *α*(*hν*) with the Tauc dependence eqn (3).^75–77^ Often the anticorrelation between *E*_0_ and *E*_U_ is linear.^75–77^ This enabled Cody^75^ to propose a concept whereby the true *E*_g_ value can be regarded as the upper limit of the experimental values of *E*_0_ obtained from eqn (3) when *E*_U_ = 0.

Analysing the *E*_0_ and *E*_U_ values in their totality is an essential tool for studying disorder. In general, the *E*_U_ and *E*_0_ values should be considered effective for heterogeneous media, since their structure differs from that of “classical” homogeneous ones. The classical Tauc relation is strictly valid for homogeneous single-phase layers. In the present work, the Cu–Si–O films represent multiphase nanocomposite systems and, after annealing, exhibit a bilayer-like structure. Therefore, the Tauc analysis is used here mainly as an effective description of the optical absorption edge evolution rather than as a strict determination of mobility gaps for individual phases.

From the XRD, Raman scattering, IR spectroscopy data, we can conclude that the fundamental absorption edge in the optical absorption spectra of the as-deposited film should be determined by absorption in (quasi)amorphous Cu_2_O and Si phases as well as intermediate Si–O–Cu amorphous phases. In addition, the absorption in these amorphous phases is superimposed by absorption in nanocrystalline Cu particles. Thus, it can be expected that the minimum value of the electronic band gap can be estimated as *E*_g_ ∼ 2.1–2.2 eV, considering the values of *E*_g_ = 2.1 eV for defect-free amorphous silicon^75^ and *E*_g_ = 2.17 eV for crystalline Cu_2_O.^68^ The electronic band gap *E*_g_ of crystalline Cu_2_O is determined by the energy distance between the valence band and first (forbidden) conduction band. Optical transitions between these electronic bands are weak due to the forbidden nature of these transitions. The electronic band gap between the valence band and the second (allowed) conduction band is 2.62 eV.^68^ The size effect can increase *E*_g_ in a-Si up to 3.22 eV.^78^ An increase in *E*_g_ can also take place in direct band gap Cu_2_O (a 0.4 eV blue shift of the first fundamental absorption peak relative to its position in a Cu_2_O single crystal was observed in Cu_2_O nanocrystalline samples with a 20 nm crystallite mean size).^79^*E*_g_ for amorphous Cu_2_O is 2.7 eV.^80^

The structural analysis data presented above suggest that, in the film annealed at 400 °C, the edge of the fundamental absorption band should be formed by absorption in the CuO and Cu_2_O phases, whereas in the film annealed at 600 °C it is formed by absorption in the CuO phase. The electronic band gap of CuO is lower compared to that of Cu_2_O: many-body *GW* calculations employing an additional onsite potential for the Cu-d orbital energies predicted for CuO an indirect electronic band gap of *E*_g_ = 1.24 eV and a direct electronic band gap of *E*_g_ = 1.46 eV.^68^ These calculations agree rather well with the optical band gap value of 1.34 eV obtained for the single crystals in ref. 81. The size-dependent band gap effect was also observed for CuO: the *E*^d^_0_ value obtained from the (*α* × *hν*)^2^*versus hν* plot was 1.64 eV in a polycrystalline CuO film with a crystal size of 18 nm,^82^ and 2.65 eV in a powder sample with a particle size of 7 nm.^83^

**Fig. 14:**
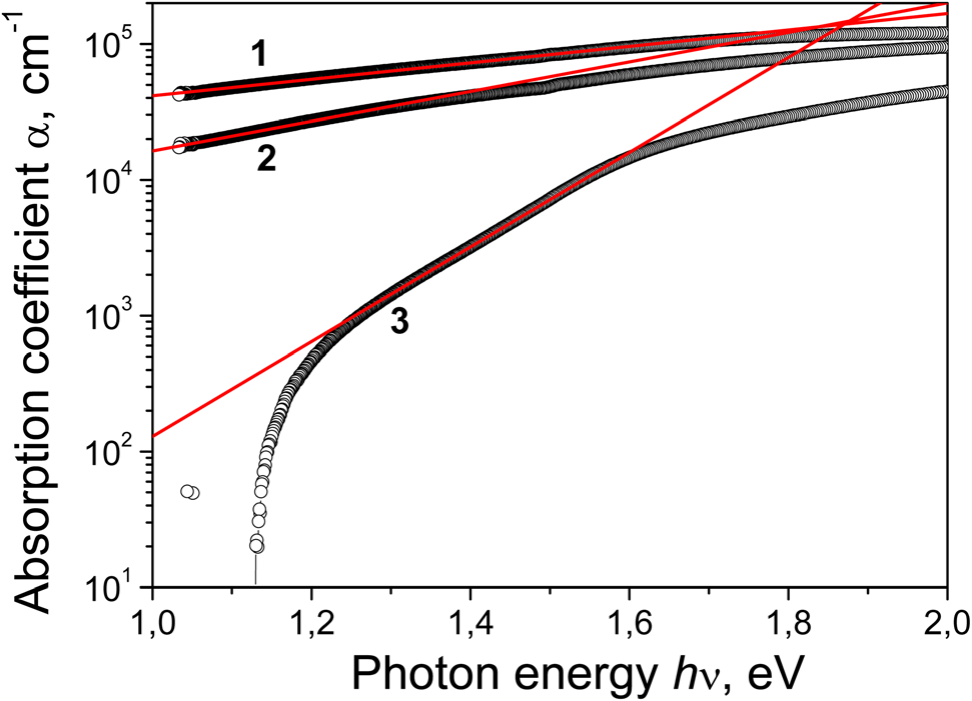
Spectral dependences of the absorption coefficient in semilogarithmic coordinates for the Cu–Si–O film before (1) and after annealing at 400 (2) and 600 °C (3).

Fig. 14 shows the *α*(*hν*) dependences in semilogarithmic coordinates in the spectral range *hν* < 2 eV for all three samples. As can be seen, these experimental plots contain quasi-linear regions. Their linear approximations are shown by the red lines. The *E*_U_ values were obtained using the slopes of these lines. For the as deposited film, the Urbach region extends up to *hν* ∼ 1.6 eV, with *E*_U_ = 0.72 eV. Annealing at 400 °C resulted in a narrowing of the range of the exponential dependence *α*(*hν*) both in the energy interval and in the values of *α*; the Urbach region extends only to *hν* ∼ 1.3 eV, with *E*_U_ = 0.40 eV. This indicates an increase in the overall ordering of the film. The Urbachian dependence is poorly fulfilled in the region of the very edge of the measured range for the film annealed at 600 °C, unlike the previous films. This may be due to the fact that eqn (1) does not fully take into account the influence of interference in the region of weak absorption. For this film, a linear region can be identified within the energy range *hν* = 1.29–1.57 eV. The slope of this region yields a value of *E*_U_ = 0.12 eV. For the as-deposited film, the large value of *E*_U_ (0.72 eV) is significantly higher than the value of *E*_U_ = 0.075 eV in pure silica glass,^72^ the *E*_U_ = 0.08 eV value in high-quality crystalline highly textured Cu_2_O and CuO films,^82,84^ and the *E*_U_ = 0.042 eV value in high-quality amorphous silicon films.^71^ Annealing reduces *E*_U_, which indicates the ordering of the film structure. The *E*_U_ value obtained after annealing at 600 °C (0.12 eV) approaches those observed in high-quality, single-phase oxide samples. This indicates a significant decrease in the defect density of CuO crystallites, since, according to XRD and Raman data, they may be responsible for the long-wavelength absorption edge. This decrease in defectiveness correlates with a decrease in the average level of microstrain in CuO crystallites (Table 2).

**Fig. 15:**
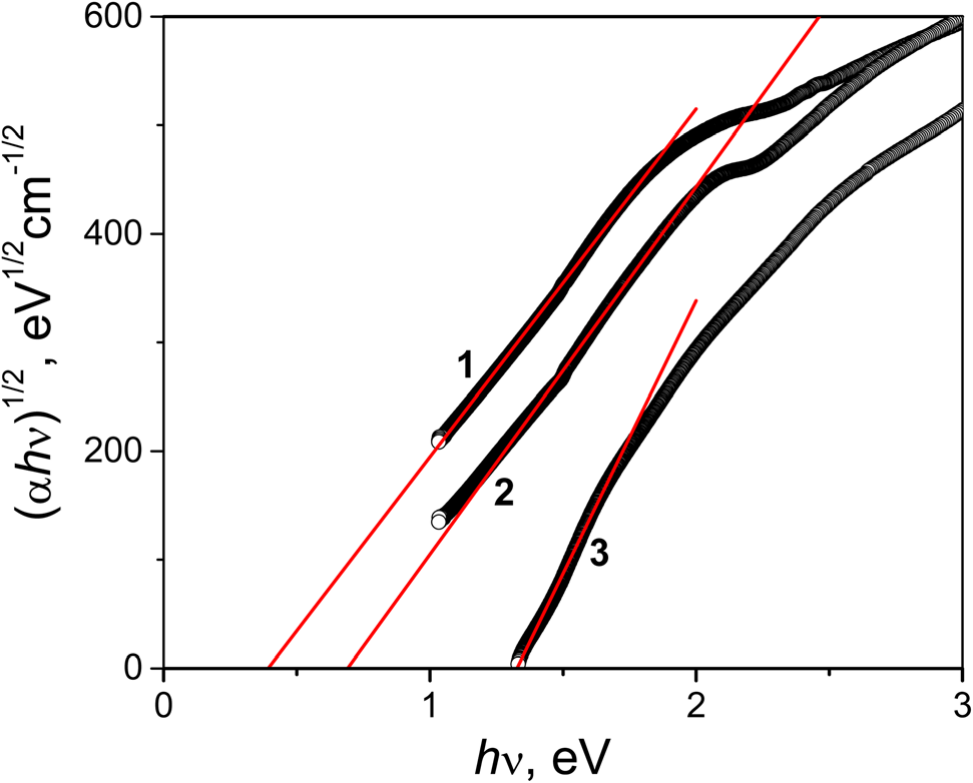
Spectral dependences of the absorption coefficient in (*αhν*)^1/2^–*hν* Tauc coordinates for Cu–Si–O film before (1) and after annealing at 400 (2) and 600 °C (3).

The *α*(*hν*) plots built in (*αhν*)^1/2^–*hν* coordinates are shown in Fig. 15. As can be seen, in this representation, all three plots at *hν* < 2 eV exhibit a quasi-linear section approximated by the red line. The Tauc optical bandgap *E*^i^_0_ is defined as the intersection of these lines with the abscissa at the *αhν* = 0 level. The *E*^i^_0_ values obtained in this way are 0.39, 0.69, and 1.325 eV for the as-deposited film and for the films annealed at 400 °C and 600 °C, respectively. As can be seen, these values are anti-correlated with the *E*_U_ values. The experimental “(*αhν*)^2/3^*vs. hν*” plot for the as-deposited Cu–Si–O film (not shown) has a narrow linear region (1.49 ≤ *hν* ≤ 1.74 eV), the extrapolation of which gives the value *E*^d^_0_ (forbidden) = 0.73 eV. This value is significantly lower than the expected one for the Cu_2_O subphase due to significant heterogeneity of the deposited film. Based on the Tauc relation for indirect band gap transitions and the energy position corresponding to the deviation from the Urbach relation, the following *E*_g_ estimates are obtained: 0.73 eV ≤ *E*_g_ ≤ 1.6 eV, 0.69 eV ≤ *E*_g_ ≤ 1.3 eV and 1.325 eV ≤ *E*_g_ ≤ 1.57 eV for the film before and after annealing at 400 and 600 °C, respectively. As can be seen, the greatest discrepancy between the *E*_g_ estimates derived from these two approaches is observed for the initial sputtered film and the smallest is observed for the film annealed at 600 °C. The obtained Tauc band gap energies (*E*^i^_0_ and *E*^d^_0_ (forbidden)) for the as-deposited film and the film annealed at 400 °C are significantly lower than the expected *E*_g_ values due to the films' pronounced heterogeneity and the presence of metallic copper inclusions. The *E*_g_ estimates, based on deviations from Urbach dependence, are closer to the expected values for these films. This is an important result. The significant decrease in the *E*_U_ value after annealing at 600 °C brings the *E*_g_ estimates derived from these two approaches much closer together. The value obtained in this case (1.325 < *E*_g_ ≤ 1.57 eV) demonstrates that the interband absorption of the film annealed at 600 °C is due to absorption in the crystalline form of CuO.

**Fig. 16:**
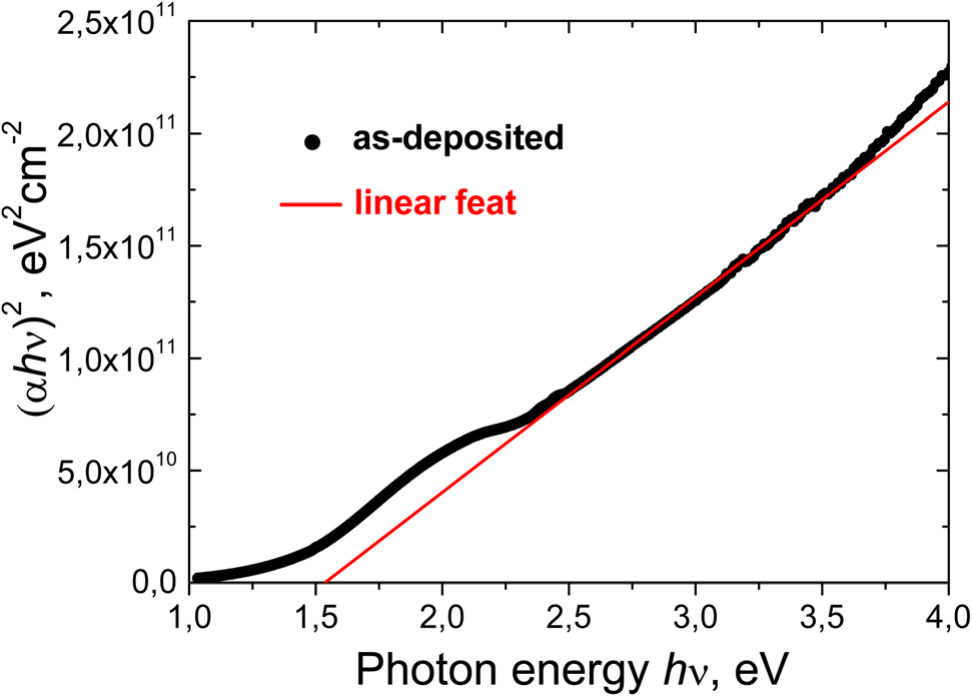
Experimental (*αhν*)^2^*versus hν* plot (circles) for the as-deposited Cu–Si–O film and linear dependence (*αhν*)^2^ = −1.335 × 10^11^ + 8.686 × 10^10^ × *hν* (red line).

Materials that exhibit indirect band gaps may also present direct transitions at higher energies. These transitions are responsible for most of the edge interband absorption in Cu_2_O.^68^ The presence of a (quasi)crystalline Cu_2_O phase in the deposited film makes it possible to expect direct allowed transitions. Fig. 16 shows the experimental “(*αhν*)^2^*vs. hν*” plot for the as-deposited Cu–Si–O film. As can be seen, in the spectral range *hν* = 2.5–3.5 eV the experimental plot for the as-deposited film is close to linear. The value *E*^d^_0_ = 1.54 eV for direct allowed transitions is obtained from the extrapolation of the linear least squares fit (red line) of (*α* × *hν*)^2^ to zero in this plot. However, due to chemical and structural heterogeneity of this film the obtained *E*^d^_0_ value is much lower than the direct electronic gap value of 2.62 eV in cuprous oxide with a perfect crystalline structure.^68^ By extrapolating the linear part of the “(*αhν*)^2^*vs. hν*” plots widely different *E*^d^_0_ values were obtained: from 2.16 in a thin film of copper oxide deposited by thermal evaporation of cuprous oxide powder^85^ to 2.38 eV in the film deposited by reactive magnetron sputtering^86^ and up to 2.51 after post-annealling of this film in air. The authors in ref. 86 found that the increase in the measured value of *E*^d^_0_ from 2.38 eV to 2.51 eV was accompanied by a decrease in the Urbach energy from 0.25 eV to 0.14 eV. The points (0.14 eV, 2.51 eV) and (0.25 eV, 2.38 eV) from ref. 86, as well as the point (0.72 eV, 1.54 eV) for our as-deposited film, are well approximated by a linear anticorrelation between *E*_U_ and *E*^d^_0_ (*R* = 0.9984). Such a linear anticorrelation at *E*_U_ = 0 eV yields an *E*^d^_0_ value of 2.77 eV.

**Fig. 17:**
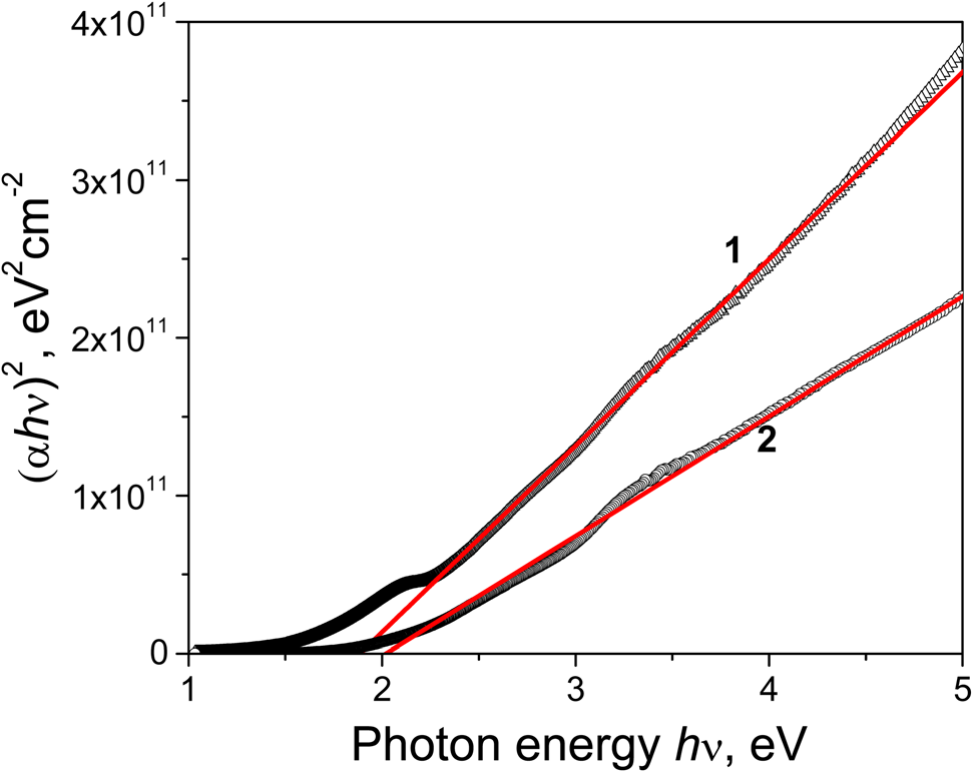
Experimental “(*αhν*)^2^*vs. hν*” plot for the Cu–Si–O films annealed at 400 (1) and 600 °C (2).

Fig. 17 shows the experimental “(*αhν*)^2^*vs. hν*” plot for the Cu–Si–O films annealed at 400 and 600 °C. The predominant contribution to the interband absorption in these films is apparently made by the CuO phase. At *T*_ann_ = 400 °C the “(*αhν*)^2^*vs. hν*” plot shows a clearly distinct straight line in the spectral range 2.3–4.5 eV, and from its intercept on the *hν* axis, the *E*^d^_0_ value is estimated to be 1.89 eV. Practically the same *E*^d^_0_ value (1.9 eV) was obtained for CuO films prepared by oxidation of a Cu_2_O film in open air at 400–500 °C.^87^ In the spectral range 2.3–5.0 eV the experimental “(*α* × *hν*)^2^*versus hν*” plot for the Cu–Si–O film annealed at 600 °C is close to linear, with *E*^d^_0_ = 2.02 eV. That is, despite the decrease in Cu_2_O phase content and the increase in CuO phase content with an increase in *T*_ann_ from 400 to 600 °C, an increase in *E*^d^_0_ occurs. Such behaviour of the determined *E*^d^_0_ can be associated with a decrease in *E*_U_ and a reduction in the size of the CuO crystallites (by almost half: from 65 to 36 nm).

For the as deposited film and the film annealed at 400 °C within the 2 eV photon energy range, the difference between the experimental and Tauc-approximated *α* values (see Fig. 16 and 17) is primarily due to plasmonic absorption in copper nanoparticles. This makes it possible to estimate their volume fraction, *q*. For this purpose, the as deposited film is considered as a composite with absorption coefficient *α*_C_ ≡ *α* consisting of isolated Cu inclusions in a continuous matrix. That is, *α* ≡ *α*_C_ ≈ *α*_M_ +*α*_P_, where *α*_M_ and *α*_P_ are the contributions of the matrix and copper nanoparticles to the total absorption coefficient of the film. From the data presented in Fig. 16, at *hν* ∼ 1.96 eV (*λ* = 633 nm), *α*_C_ = 1.196 × 10^5^ cm^−1^, *α*_M_ = 9.77 × 10^4^ cm^−1^, and *α*_P_ = 2.185 × 10^4^ cm^−1^. Accordingly, for the absorption indices, *k* = (*λ* × *α*)/4π, we have *k*_C_ = 0.602, *k*_M_ = 0.49, and *k*_P_ = 0.112. Optical characteristics of such composite media can be described by the Maxwell Garnett electrodynamic theory of effective medium^88^ to determine the volume concentration of copper particles. For our case the Maxwell Garnett formula can be written as4
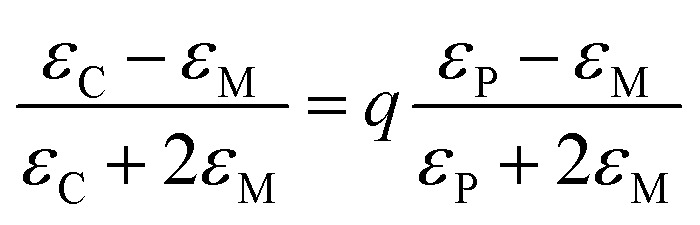
where *ε*_C_, *ε*_M_ and *ε*_P_ and are the complex optical dielectric functions (*ε*_i_ = [*n*_i_ − i*k*_i_]^2^) of the composite, matrix and included particles (*n*_i_, *k*_i_ – refractive and absorption indexes).

In the region of the absorption band caused by plasmonic absorption in Cu nanoparticles the value of *T*/*T*_s_ is ∼2.5%. On the one hand, this allows us to determine the absorption coefficient *k* of the film with sufficient accuracy using eqn (1) since the contribution of interference in this case is very small. On the other hand, reflection from such a film practically corresponds to the case of reflection from two semi-infinite media, one of which is the deposited film and the other is air, *i.e.*5
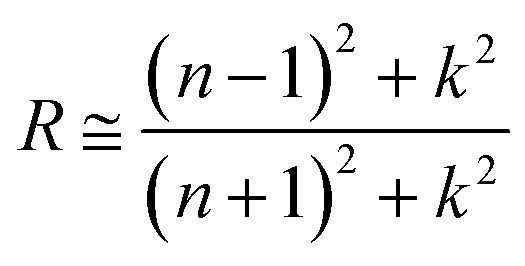
where *n* and *k* are the refractive and absorption indexes of the film. Having the measured value of *R* and the value of *k* calculated using eqn (1), from eqn (5) we can determine the value of *n*, which for the deposited film at *λ* = 633 nm is *n* = 3.23. Thus, using the values of *n* and *k* obtained using eqn (1) and eqn (5), we know the value of *ε*_C_ ≡ *ε* = (*n* − i*k*)^2^.

In the deposited film, the average size of copper particles is 15 nm, which is tens of times smaller than the electron mean free path in bulk Cu.^89^ As a result, the optical constants of small metal particles differ from the optical constants of bulk metal and depend on their size. To obtain the values of optical constants of such particles, the values of optical constants for the bulk metal, corrected for the effect of a reduced mean free path of the conduction electrons in small particles, are used.^90^ In the quasi-classical approximation, this effect is associated with the limitation of the electron mean free path by the particle surface. For spherical particles, the mean free path is equal to their radius. More accurate quantum mechanical calculations of the influence of size effects on the optical properties of metallic particles have also been performed. However, a number of experimental studies have shown that the quasi-classical approximation describes the size dependence of the optical constants for small (less than 20 nm) copper particles no less adequately.^91^ Within this approximation, the optical dielectric function of a metallic particle is expressed as^90^6
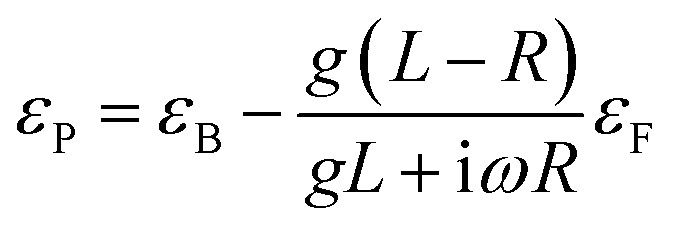
where *ε*_B_ = *ε*_1B_ − i*ε*_2B_ is the optical dielectric function of bulk metal. *ε*_F_ = [*ω*_p_^2^*F*]/[*ω*(*ω* − i*g*)] is the contribution of free electrons to the optical dielectric function of bulk metal (*ω*_p_^2^ = *Ne*^2^/*ε*_0_*m*, *g* = *ε*_0_*ω*_p_^2^*F*/*σε*_0_, *L* = (2*E*_F_ mg^−2^)^1/2^. Here *g*—damping parameter; *L*—mean free path in the bulk metal; *R*—the radius of the particles; *F*—the fraction of free electrons that is effective; *ω*_p_—plasma frequency; *E*_F_—Fermi energy). When calculating the optical dielectric function of copper particles the values of *ε*_B_ are taken from ref. 92 and *R* = *D*/2, where *D* is the crystallite size of copper inclusions determined from the diffractograms of the deposited film using the Williamson–Hall method.^43–47^

From eqn (4) the volume fraction of metal particles can be expressed as7
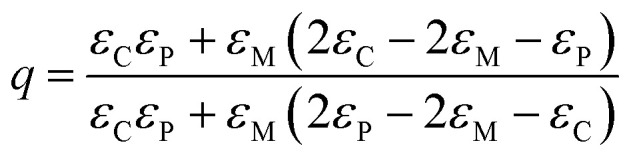


In eqn (7) in addition to the volume fraction of metal particles *q*, the unknown quantity is the refractive index of the matrix *n*_M_, which is included in the quantity *ε*_M_ as *ε*_M_ = [*n*_M_ − i*k*_M_]^2^. The values of *q* and *n*_M_ are obtained by solving a pair of equations given by the real and imaginary parts of eqn (7) [*q* = *q*_r_ − i*q*_i_] with the condition that *q*_i_ = 0, since the volume fraction is geometrical in nature and should be real. The resulting value is *q* = 0.03.

Thus, using the reflectance–transmittance spectroscopy technique, thorough research into the evolution of absorption spectra in the near band and inter-band absorption regions of heterogenous multiphase nanocomposite Cu–Si–O films as a result of annealing at *T*_ann_ ≤ 600 °C was performed. Based on the obtained spectra for the absorption coefficient *α* in the energy range of 1.03–6.53 eV, the optical band gap or Tauc band gap *E*_0_ and the width of the exponential absorption tail (Urbach tail energy) *E*_U_, which together are an important tool for studying heterogeneity and (dis)ordering of complex materials, were estimated. The behaviour of these parameters with increasing annealing temperature indicates an increase in the overall ordering of the film up to 600 °C. Absorption bands caused by plasmonic absorption in Cu nanoparticles have been observed in both the as-deposited film and the film annealed at 400 °C. Using Maxwell Garnett theory and considering the influence of size effects on the optical constants of copper, the volume fraction of copper nanoparticles in the as-deposited film was estimated to be 3%. Annealing at 600 °C leads to the disappearance of this plasmonic absorption band, which is consistent with the results of XRD studies.

## 4 Conclusions

The films obtained by ion-plasma sputtering of a combined Cu/Si target in an Ar/O_2_ atmosphere are uniform nanocomposites consisting of a heterogeneous mixture of silicon and copper suboxides and oxides with nano- and dispersed inclusions of copper and silicon. The presence of silicon and copper oxide and suboxide phases was confirmed using XPS, FTIR, and XRD spectroscopy. The crystalline Cu nanoinclusions were identified by XRD, and the amorphous Si nanoinclusions were identified by Raman spectroscopy. The size of Cu nanocrystallites was estimated to be ∼15 nm.

According to the results of SEM studies, it was found that depending on the annealing temperature, significant transformations occur in both the internal structure of the films and their surface morphology. The macrostructure of the film becomes bilayered with the presence of island formations, crystal grains, and pores. The XPS and D-SIMS methods revealed a redistribution of Si and Cu atoms across the depth of nanocomposite films, and it was also shown that an increase in temperature leads to the release of copper atoms to the surface, additional oxidation of silicon, the formation of porous structures and the formation of CuO and Cu–Si–O phases. Heat treatment causes successive phase transformations. Cu and Cu_2_O oxidize to CuO up to 600 °C. FTIR studies showed that the intensity of the Si–O bands in the as-deposited film significantly exceeds that of the Cu–O bands. This indicates the possible presence of copper atoms or clusters in the silicon oxide matrix.

According to the results of combined reflection–transmission spectroscopy, a plasmon absorption band corresponding to Cu nanoparticles was detected in the as-deposited film. The volume fraction of nanoparticles, estimated using the Maxwell Garnett theory, is 3%. The absorption coefficient spectra were used to determine parameters such as the Tauc optical band gap (*E*_0_) and the Urbach tail energy (*E*_U_), which characterise the heterogeneity and ordering of the film. The electronic band gap of the narrowest-band-gap subphase was estimated. The change of these parameters with the annealing temperature indicates an improvement in ordering up to 600 °C.

The results demonstrate that the structure and physicochemical properties of Cu–Si–O films can be effectively tuned over a wide range, which makes these films promising for applications in resistive switching structures, sensing systems, and dielectric layers for micro- and nanoelectronics.

## Author contributions

Oleh Bratus: conceptualization, project administration, methodology, resources, data curation, investigation, formal analysis, visualization, writing—original draft, and writing—review and editing. Antonina Kykot: investigation, visualization, and writing—original draft. Mykola Sopinskyy: investigation, formal analysis, visualization, writing—original draft, and writing—review and editing. Pavels Onufrijevs: funding acquisition, writing—review and editing. Oleksandr Gudymenko: investigation, formal analysis, visualization, and writing—original draft. Larysa Khomenkova: investigation, visualization, writing—original draft, and writing—review and editing. Volodymyr Yukhymchuk: investigation, visualization, and writing—original draft. Tomash Sabov: investigation, visualization and writing—original draft. Oleksandr Oberemok: investigation, visualization, and writing—original draft. Anton Semeniuk: investigation and writing—review and editing. Dmytro Kysil: investigation and visualization. Anatoliy Evtukh: supervision, funding acquisition, writing—review and editing.

## Conflicts of interest

There are no conflicts to declare.

## Data Availability

Due to the large volume of raw data obtained during this study, the data will be provided upon reasonable request.
